# Safety and efficacy of p38 mitogen-activated protein kinase inhibitors (MAPKIs) in COPD

**DOI:** 10.3389/fphar.2022.950035

**Published:** 2022-09-28

**Authors:** Haichuan Yu, Xiaojie Su, Ting Lei, Lu Zhang, Zhouzhou Feng, Chuchu Zhang, Meng Zhang, Yalei Wang, Xinlong Chen, Jian Liu

**Affiliations:** ^1^ The First Clinical Medical College of Lanzhou University, Lanzhou, China; ^2^ Intensive Care Unit, The First Hospital of Lanzhou University, Lanzhou, China

**Keywords:** COPD, systematic review, meta-analysis, p38 MAPK, safety, efficacy

## Abstract

**Introduction:** Chronic inflammation is the core mechanism of the development of chronic obstructive pulmonary disease (COPD). Corticosteroid resistance in COPD limits its anti-inflammatory potency. p38 MAPKIs were suggested as an alternative to corticosteroids despite the fact that there is currently no systematic review evaluating existing evidence.

**Methods:** This randomized controlled trials (RCT)-based systematic review with meta-analysis was conducted following the PRISMA statement. RCTs were searched and screened from 8 databases. Three types of data, including basic information of included studies, pre-defined outcome data, and quality assessment information were extracted. Pooling values and associated 95 % confidence intervals were deemed as statistically significant only when two-tailed *p* values were smaller than 0.05.

**Results:** This study included 10 RCTs with a total population of 1,751 [age, mean (SD) = 64.39 (8.06)]. Safety and several efficacy indicators of lung function, inflammatory biomarkers, and quality of life were meta-analyzed. Despite the improvement of post-bronchodilator-forced vital capacity (FVC), no difference between p38 MAPKIs and placebo was found in both safety and efficacy.

**Conclusion:** Compared with placebo, p38 MAPKIs are safe but did not show any significant effects in the COPD population. Results of this study should be regarded with caution due to the small number of included studies and heterogeneity from combining different p38 MAPKIs as a whole.

**Systematic Review registration:** PROSPERO #CRD42022302890.

## Introduction

Chronic obstructive pulmonary disease (COPD) is a chronic and progressive disease characterized by irreversible airflow limitation, frequent exacerbation, physical intolerance, and impairment of the quality of life (Gold Science Committee, 2021). It has become the third leading cause of death globally (GBD Chronic Respiratory Disease Collaborators, 2020). Systemic inflammation is one of the key mechanisms of the development of COPD (Gan et al., 2004; Garcia-Rio et al., 2010). Meanwhile, corticosteroids, being the most widely used anti-inflammatory drugs, have little effect and are not recommended as monotherapy in COPD patients (Barnes, 2013; Gold Science Committee, 2021). Several pathways were examined and several medications are under development to better understand the mechanism of corticosteroid resistance and pave the way for anti-inflammation therapy (Hakim et al., 2012).

The p38 mitogen-activated protein kinase (p38 MAPK) is a serine/threonine-protein kinase that has been discovered to be an important facilitator in the expression of various inflammatory agents (Lee et al., 1999). It has been observed that activating p38 MAPK causes the hyperphosphorylation of nuclear factor kappa-light-chain-enhancer of activated B cells (NF-*κ*B), inhibition of the glucocorticosteroid receptor, and a reduction in corticosteroid-induced MAPK-1, ultimately leading to corticosteroid resistance (Chung, 2011). p38 MAPK over-expression has also been found in lung tissue and correlated with poorer lung function in the COPD population (Renda et al., 2008; Gaffey et al., 2013). Moreover, in the rat model, p38 MAPKIs showed a promising effect on inhibiting airway inflammation (Escott et al., 2000; Duan et al., 2005). Hence, a hypothesis emerged that p38 MAPKIs might slow down the progression of the disease and improve the prognosis of patients with COPD by suppressing inflammation (Banerjee et al., 2012; Norman, 2015).

After long-term screening, the safety of the existing p38 MAPKIs was confirmed in the majority of published studies; however, evidence of p38 MAPKIs’ efficacy remains highly controversial (Chopra et al., 2008). In brief, there are two contrasting schools of view and we made a table to summarize existing evidence (Table 1). One school of view believes that the use of p38 MAPKIs in COPD patients has an important exploratory value in terms of its efficacy in improving lung function, reducing inflammation levels, and decreasing the frequency of acute exacerbations. The other school of view is that p38 MAPKIs have failed to play a role in COPD patients and thus further studies may be meaningless. As there is still no systematic review to conclude these mixed evidence, we conducted this systematic review and meta-analysis to explore the efficacy and safety of p38 MAPKI in patients with COPD.

**TABLE 1:** The controversies in the efficacy of p38 MAPKI on COPD summarized from existing reports of RCTs.

Indicators	Pros			Cons
	Study	Perspectives	Study	Perspectives
Lung function	Marks-Konczalik et al. (2015)	Losmapimod can improve lung function in patients whose blood eosinophils >2%	Patel et al. (2018)	AZD-7624 cannot improve lung function
	Charron et al. (2017)	RV-568 can improve FEV1/RV.	Watz et al. (2014)	Losmapimod has little positive effect on lung function
	Lomas et al. (2012)	Losmapimod can improve lung function according to the results of cluster analysis		
	MacNee et al. (2013)	PH-797804 can improve FEV1		
	Strâmbu et al. (2019)	Acumapimod can transiently improve FEV1		
	Watz et al. (2014)	Losmapimod can improve FEV1		
Exacerbations	Marks-Konczalik et al. (2015)	Losmapimod can lower the exacerbation rate in patients whose blood eosinophils ≤2%	Pascoe et al. (2017)	Losmapimod cannot lower the exacerbation rate in patients whose blood eosinophils ≤2%
Inflammatory biomarkers	Marks-Konczalik et al. (2015)	Losmapimod can transiently lower the level of hsCRP and fibrinogen	Patel et al. (2018)	AZD-7624 cannot lower the level of observed inflammatory biomarkers
	Charron et al. (2017)	RV-568 can lower the serum/sputum level of oxidative stress biomarker MDA.		
	Lomas et al. (2012), Fisk et al. (2018)	Losmapimod can lower the level of fibrinogen		
	Singh et al. (2010)	SB-681323 can lower the level of TNF-α and pHSP.		
	Watz et al. (2014)	Losmapimod can lower the level of hsCRP and fibrinogen		

FEV1, forced expiratory volume in 1 s; FEV1%pred, percent of forced expiratory volume in 1 s on prediction; RV, residual volume; hsCRP, high sensitivity C-reactive protein; MDA, malondialdehyde; TNF-α, tumor necrosis factor-α; pHSP, phosphorylated heat shock protein. **Notes:** Losmapimod, RV-568, PH797804, acumapimod, SB-681323 mentioned in this table are all p38 MAPKIs.

## Methods

This systematic review and meta-analysis followed the Preferred Reporting Items for Systematic Review and Meta-analyses (PRISMA) statements (PRISMA checklist Supplementary Material) (Page et al., 2021). The protocol of this study was registered in the International Prospective Register of Systematic Reviews (PROSPERO #CRD42022302890) (Yu et al., 2022).

### Literature search

Eight databases including PubMed, Embase, Web of Science (WOS), China National Knowledge Infrastructure (CNKI), Chinese biomedical literature service system (SinoMed), ClinicalTials.gov, and the International Clinical Trials Registry Platform (ICTRP) were searched. “p38 Mitogen-Activated Protein Kinases” and “Pulmonary Disease, Chronic Obstructive” were the major terms to build the search strategy (Search details Supplementary Material).

### Literature screening

This study only included randomized controlled trials (RCTs). The included studies all focused on the efficacy and safety of p38 MAPKI in patients with COPD, regardless of the specific type of drug or how the drug was administered. Two independent reviewers carried out the screening process, while a third experienced reviewer mediated a discussion to combine the screening results.

### Data extraction and quality assessment

Preliminarily, a data-extraction chart containing the characteristics of the studies, risk of bias assessment, and outcomes was designed. Data extraction and quality assessments were conducted by 2 reviewers separately, and the article author held a discussion to combine different judgments. The risk of bias was assessed utilizing the Cochrane Risk Of Bias (ROB) Tool for RCTs. Our study includes information on the efficacy and safety of administering p38-MAPKI. If indicators were reported at multiple time points, the longest follow-up data would be collected for safety and the most significantly changed data would be collected for efficacy. The outcomes were all analyzed as the primary outcomes, no matter the manner of the meta-analysis or narrative synthesis.

If available, the missing data were estimated using Review Manager (version 5.4), Engauge Digitizer (version 4.1), graphical data extraction software, and online calculator StatsToDo (StatsToDo, 2020).

Moreover, after the completion of the statistical analysis, GRADEpro GDT was used to evaluate the overall quality of evidence (Inc. MUaEP, 2022).

### Statistical analysis

An outcome must be extracted from at least 2 different studies to be included in meta-analysis; otherwise it would be a narrative synthesis. For continuous data such as lung function and inflammatory biomarkers, the standardized mean difference (SMD) instead of the mean difference was calculated to compensate for the differences due to the different measurements in different studies; for dichotomous data such as the incidence of adverse events, the risk ratio (RR) was calculated; and for both types of data, the 95 % credit interval (CI) was calculated at the same time. Heterogeneity was quantified by using the *I*-square (*I* ^*2*^) test before the pooling procedure: a randomized effect model (REM) was used for pooling if *I* ^*2*^ > 50 %, otherwise a fixed-effect model (FEM) was used. Sensitivity analysis was conducted if *I* ^*2*^ > 50 %, and a subgroup analysis was conducted if necessary. Publication bias would be tested by using funnel plot and Egger’s test if the number of the included studies was more than 10 (Egger et al., 1997). Only when two-tailed *p* values were smaller than 0.05 could the pooling estimations be deemed as statistically significant.

R (version 4.1.1, meta package [version 5.2-0]) was used to compute all statistical analyses.

## Results

The literature search yielded 1,077 articles, from which 10 RCTs with a total population of 1,751 [age, mean (SD) = 64.39 (8.06)] were included in our final analysis (Table 2). (Singh et al., 2010; Lomas et al., 2012; MacNee et al., 2013; Watz et al., 2014; Pfizer PCgCC, 2016; Charron et al., 2017; Pascoe et al., 2017; Fisk et al., 2018; Patel et al., 2018; Strâmbu et al., 2019) After reading the entire article, one study that would have met the inclusion criteria was eliminated due to a lack of targeted outcomes (Marks-Konczalik et al., 2015). Losmapimod was the focus of four of the five p38 MAPKI studies, whereas RV 568/AZD 7624/PH 797804/PF-03715455/SB-68-1323/Acumapimod were all investigated by only one. More characteristics of the included studies are demonstrated in Table 3.

**TABLE 2:** PRISMA 2020 flow diaphragm.

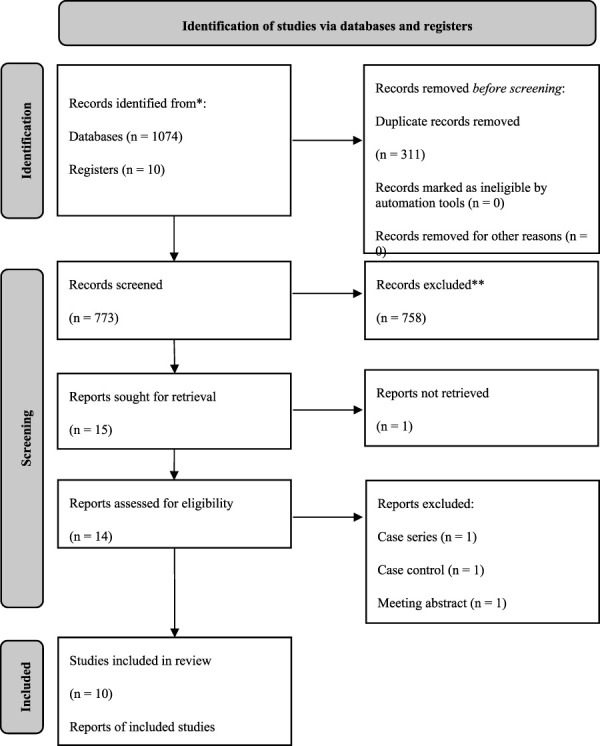

**TABLE 3:** Characteristics of the included studies.

Study	Region	Population	Age (years)	Gender (F/M)	FEV1%pred	Intervention
Charron et al. (2017)	United Kingdom	30	62.77, 5.47	16, 14	64.74, 9.92	RV 568 (50/100ug, Inhal., qd, 2W)
Fisk et al. (2018)	United Kingdom	73	67.51, 7.47	22, 51	51.03, 20.46	Losmapimod (7.5mg, po, bid, 16W)
Lomas et al. (2012)	Multi	200	62.00, 6.50	46, 154	64.50, 10.50	Losmapimod (7.5mg, po, bid, 12W)
MacNee et al. (2013)	Multi	230	64.10, 7.19	67, 163	53.63, 12.09	PH 797804 (0.5/3/6/10mg, po, qd, 6W)
NCT02366637, Pfizer PCgCC, (2016)	United Kingdom	13	62.00, 6.50	8, 5	NA	PF-03715455 (680mg, Inhal, bid, 4W)
Pascoe et al. (2017)	Multi	190	65.48, 7.56	59, 131	47.95, 15.83	Losmapimod (15mg, po, bid, 26-52W)
Patel et al. (2018)	Multi	213	64.81, 8.70	76, 137	44.50, 15.22	AZD 7624 (1.0mg, Inhal., single dose)
Singh et al. (2010)	United Kingdom	17	63.2, NA	5, 12	57.1, NA	SB-681323 (7.5/25mg, po, single dose)
Strâmbu et al. (2019)	Multi	183	62.00, 7.90	37, 146	48.80, 12.74	Acumapimod (20/40/75mg, po, single/repeat dose, 10D)
Watz et al. (2014)	Multi	602	65.22, 8.62	191, 411	45.72, 14.46	Losmapimod (2.5/7.5/15mg, po, bid, 24W)

FEV1% pred, percent of forced expiratory volume in 1 s on prediction; p38 MAPKI, p38 mitogen activated protein kinase; Inhal., inhalation; po, per os; qd, once daily; bid, twice daily; W, weeks; D,days. **Notes:** all continuous data were presented as “mean, standard deviation”; all dichotomous data were presented as “number".

Given that all studies were registered online with well-designed protocols and that their reports basically conformed to relevant protocols, the risk of bias of the included studies could be graded as “low” overall (see Table 4). A sensitivity analysis was conducted for all the outcomes whose *I* ^*2*^ > 50 %, and no outcomes’ stability was significantly influenced by the heterogeneity. Publication bias was not conducted since the quantity of the included studies of every outcome did not meet the standard (a maximum of 10 included trials for one outcome). By using GRADEpro GDT, the certainty was graded as “high” for most analyzed outcomes; heterogeneity is the main reason that impairs the certainty of evidence.

**TABLE 4:** Summary of the risk of bias.

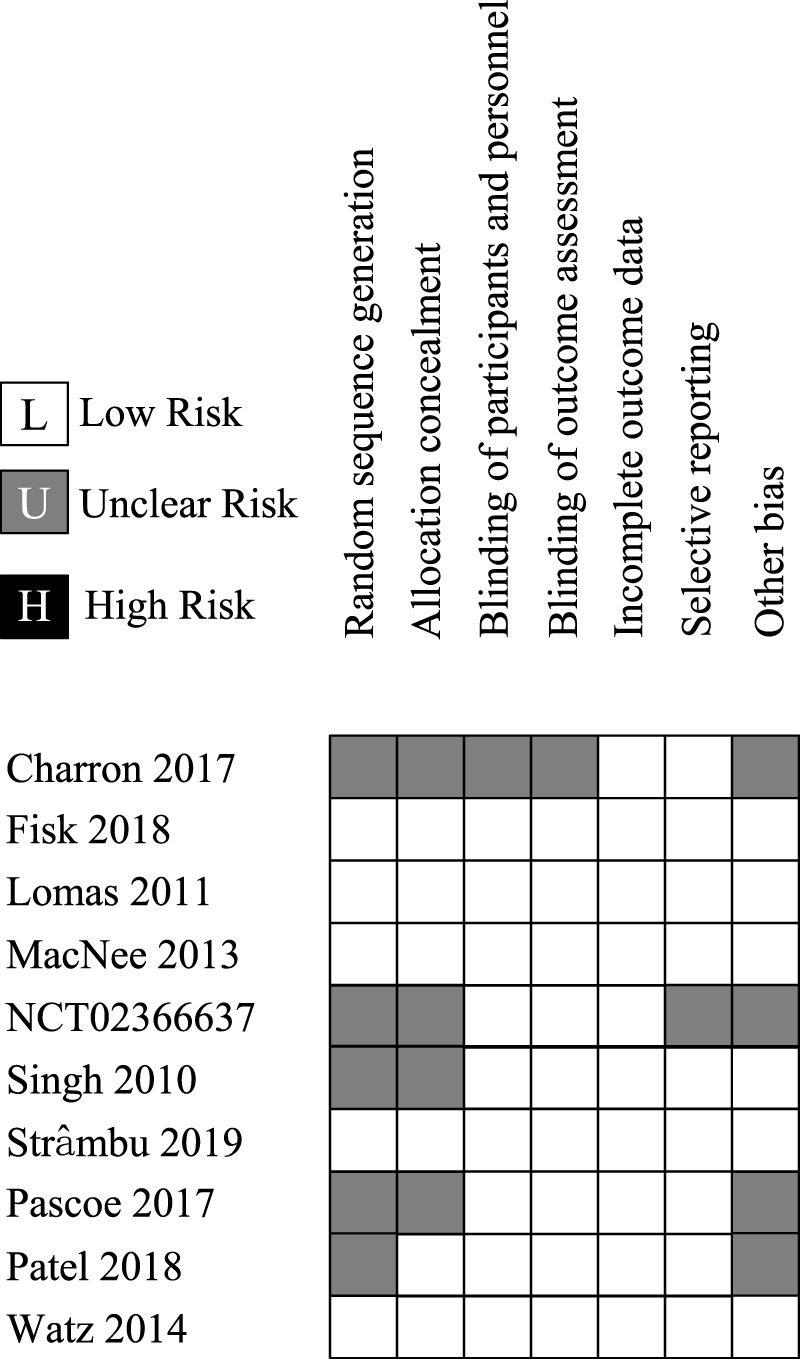

Furthermore, we found RV-568 focused by Charron et al. (2017) targeted 3 kinases (p38 α/γ and hematopoietic kinase). We therefore re-analyzed the data with the exception of *Charron et al.*, but no result was changed.

All pooling estimations are presented in Table 5. The detailed analysis process and results are presented in the Supplementary Material.

**TABLE 5:** Summary of findings.

Domain	Outcomes	Population	*I* ^*2*^ (%)	Pooling model	Pooling estimation [95% CI]	*p* value	GRADE certainty
Safety	Any AE	1134 (6 RCTs)	30	Fixed effect	RR; 1.09 [0.96, 1.24]	0.16	High
	Severe AE	1287 (6 RCTs)	64	Random effect	RR; 1.34 [0.66, 2.72]	0.42	Moderate
	ECOPD	1347 (7 RCTs)	41	Fixed effect	RR; 1.06 [0.84, 1.33]	0.63	High
	Neuro-AE	1593 (9 RCTs)	41	Fixed effect	RR; 0.76 [0.55, 1.05]	0.10	High
	DENT AE	1593 (9 RCTs)	35	Fixed effect	RR; 1.13 [0.82, 1.55]	0.47	High
	Other RI	1163 (7 RCTs)	35	Fixed effect	RR; 1.22 [0.86, 1.73]	0.27	High
	CV AE	791 (7 RCTs)	0	Fixed effect	RR; 1.69 [0.69, 4.11]	0.25	High
	Digestive AE	920 (4 RCTs)	0	Fixed effect	RR; 1.35 [0.81, 2.25]	0.25	High
	Urinary AE	905 (3 RCTs)	14	Fixed effect	RR; 3.29 [0.97, 11.20]	0.06	High
	MP	1332 (6 RCTs)	0	Fixed effect	RR; 0.93 [0.70, 1.25]	0.65	High
Efficacy	Pre-BD FEV1	711 (4 RCTs)	64	Random effect	SMD; 0.23 [-0.27, 0.73]	0.38	Moderate
	Post-BD FEV1	1190 (6 RCTs)	0	Fixed effect	SMD; 0.11 [-0.01, 0.24]	0.08	High
	Pre-BD FVC	683 (3 RCTs)	22	Fixed effect	SMD; 0.07 [-0.10; 0.23]	0.43	High
	Post-BD FVC	1075 (4 RCTs)	38	Fixed effect	SMD; 0.14 [0.01, 0.27]	0.03	High
	FEV1/FVC	709 (2 RCTs)	96	Random effect	SMD; 0.42 [−0.41, 1.25]	0.32	Moderate
	TLC	651 (2 RCTs)	76	Random effect	SMD; -0.08 [−0.45, 0.30]	0.68	Moderate
	IC	846 (3 RCTs)	70	Random effect	SMD; 0.07 [−0.24, 0.39]	0.65	Moderate
	SGRQ	905 (4 RCTs)	21	Fixed effect	SMD; -0.04 [−0.18, 0.10]	0.61	High
	hsCRP	711 (2 RCTs)	61	Fixed effect	SMD; -0.22 [−0.38; -0.05]	0.09	Moderate
	hsCRP ratio	241 (2 RCTs)	0	Fixed effect	SMD; -0.08 [−0.34; 0.17]	0.52	High
	fibrinogen	614 (2 RCTs)	86	Random effect	SMD; -0.37 [−0.92; 0.18]	0.19	Moderate

CI, confidence interval; RR, risk ratio; SMD, standardized mean difference; AE, adverse events; ECOPD, exacerbation of COPD; DENT, dental symptoms or symptoms occurred in ear, nose or throat; RI, respiratory infection; CV, cardiovascular; MP, musculoskeletal pain; BD, bronchodilator; FEV1: forced expiratory volume in 1 s; FVC: forced vital capacity; TLC: total lung capacity; IC: inspiratory capacity; SGRQ: St Georges’ respiratory questionnaire; hsCRP: high sensitivity C-reactive protein.

### Safety

All 10 studies assessed the safety of administrating p38 MAPKI. No increased risk was observed in the incidence of 1): any adverse events [reported by 6 studies of 1,134 included objects, *I* ^*2*^ = 30 %, FEM, RR (95 % CI) = 1.09 (0.96, 1.24), *p* = 0.16, high certainty], 2): severe adverse events [reported by 6 studies of 1,287 included objects, *I* ^*2*^ = 64%, REM, RR (95 % CI) = 1.34 (0.66, 2.72), *p* = 0.42, moderate certainty], 3): exacerbations of COPD [reported by 7 studies of 1,347 included objects, *I* ^*2*^ = 48%, FEM, RR (95 % CI) = 1.06 (0.84, 1.33), *p* = 0.63, high certainty], 4): neurological or sensory abnormalities [reported by 9 studies of 1,593 included objects, *I* ^*2*^ = 41 %, FEM, RR (95 % CI) = 0.76 (0.55, 1.05), *p* = 0.10, high certainty], 5): dental symptoms or symptoms that occurred in the ear/nose/throat [reported by 9 studies of 1,593 included objects, *I* ^*2*^ = 35 %, FEM, RR (95 % CI) = 1.13 (0.82, 1.55), *p* = 0.47, high certainty], 6): respiratory infections [reported by 7 studies of 1,163 included objects, *I* ^*2*^ = 35 %, FEM, RR (95 % CI) = 1.22 (0.86, 1.73), *p* = 0.27, high certainty], 7): cardiovascular events [reported by 7 studies of 791 included objects, *I* ^*2*^ = 0%, FEM, RR (95 % CI) = 1.69 (0.69, 4.11), *p* = 0.25, high certainty], 8): digestive adverse events [reported by 4 studies of 920 included objects, *I* ^*2*^ = 0 %, FEM, RR (95 % CI) = 1.69 (0.69, 4.11), *p* = 0.25, high certainty], and 9): urinary infections [reported by 3 studies of 905 included objects, *I* ^*2*^ = 14 %, FEM, RR (95 % CI) = 3.29 (0.97, 11.20), *p* = 0.06, high certainty] and musculoskeletal pain [reported by 6 studies of 1,332 included objects, *I* ^*2*^ = 0 %, FEM, RR (95 % CI) = 0.93 (0.70, 1.23), *p* = 0.65, high certainty].

### Efficacy

Spirometrical indicators were used to describe the lung function in 6 included studies, but only modest improving trends were observed among these results despite FEV1 and FVC. Since the spirometry can be conducted before or after administrating bronchodilator (pre/post BD), we analyzed such parameters separately. And no difference was found in most indicators PreBD FEV1 was reported in 4 studies with 711 patients included, no difference was found between the p38 MAPKI group and Control group [*I* ^*2*^ = 64%, REM, SMD (95% CI) = 0.13 (−0.03, 0.30), *p* = 0.38, moderate certainty]. PostBD FEV1 was reported in 6 studies with a total population of 1,190, but no statistically significant difference was identified in the pooled data [*I* ^*2*^ = 0 %, FEM, SMD = 0.11, 95 % CI (−0.01, 0.24), *p* = 0.08, high certainty]. Meanwhile, slight difference between p38MAPKIs and placebo was found in FVC (only in the postBD subgroup) [preBD, 3 studies with 683 included patients, *I* ^*2*^ = 22 %, FEM, SMD (95 % CI) = 0.07 (−0.09, 0.25), *p* = 0.35, high certainty; postBD, 4 studies with 1,075 included patients, *I* ^*2*^ = 38 %, FEM, SMD (95 % CI) = 0.14 (0.01, 0.27), *p* = 0.04, high certainty]. Additionally, there is no statistically significant difference in the remaining spirometrical indicators reported by at least two studies, which were FEV1/FVC [reported by 2 studies of 709 included objects, *I* ^*2*^ = 96 %, REM, SMD (95 % CI) = 0.42 (−0.41, 1.25), *p* = 0.32, moderate certainty], IC [reported by 3 studies of 846 included objects, *I* ^*2*^ = 70 %, REM, SMD (95 % CI) = 0.07 (−0.24, 0.39), *p* = 0.01, moderate certainty], RV [reported by 2 studies of 651 included objects, *I* ^*2*^ = 86 %, REM, SMD = −0.02, 95 % CI (−0.50, 0.47), *p* < 0.01], and TLC [reported by 2 studies of 651 included objects, *I* ^*2*^ = 76 %, REM, SMD (95 % CI) = −0.08 (−0.45, 0.30), *p* = 0.68, moderate certainty].

St. George’s Respiratory Questionnaire (SGRQ) is the most popular measurement of the quality of life in patients with COPD. It is widely used because of its ability to simultaneously assess symptom severity, activity limitation, and social or psychological impairment caused by respiratory diseases. The SGRQ scores reported by 4 studies including 905 patients were meta-analyzed in this systematic review, and no significant effect was detected [*I* ^*2*^ = 21 %, FEM, SMD (95 % CI) = −0.04 (−0.18, 0.10), *p* = 0.29].

Inflammatory biomarkers were reported in 6 included studies, but most indicators were reported in different manners, except the FIB, hsCRP, and hsCRP ratios. There was no statistically significant difference between these three indicators that can be included in the meta-analysis; however, there was a slightly decreasing trend in their meta-analysis pooling estimations. The geometric mean of the ratio to the baseline value was reported by Fisk et al. (2018). They found merely a decreasing trend throughout the whole study period in FIB and hsCRP. In a study by Lomas et al. (2012), all results were reported by the ratio of effect of the intervention group/placebo group. They discovered no difference between losmapimod and placebo in the sputum neutrophil count and most blood biomarkers, including interleukin 6 (IL-6), interleukin 8 (IL-8), C-reactive protein (CRP), matrix metalloprotein 9 (MMP-9), Clara cell secretory protein 16 (CC-16), and surfactant protein D (SP-D). Meanwhile, statistically significant reduction was observed in plasma fibrinogen [ratio of effect of losmapimod/placebo = 0.89, 95 % CI (0.83, 0.96); *p* = 0.002] and the result of systematic inflammation cluster of the *O’Brien* multivariate analysis (*p* = 0.019). MacNee et al., (2013) reported the ratio of log mean values (PH 797804/placebo). After 6 weeks of treatment, they found a significant decrease in hsCRP in 3 intervention groups [for groups with a 3 mg dose, 0.633 (*p* = 0.033); 6 mg, 0.588 (*p* = 0.011); and 10 mg, 0.594 (*p* = 0.021)], while no difference was found in the CC-16, IL-6, SP-D, and FIB groups. Moreover, Patel et al. (2018) reported no difference between AZD 7624 and placebo in hsCRP (*p* = 0.44), IL-6 (*p* = 0.09), and MIP-1β (*p* = 0.20). Since no variance in their means was provided, the results of the study cannot be included in our meta-analysis.

Ergometric indicators can reflect the activity limitations of patients. They were reported only in the study of Watz et al. (2014), with no significant observable difference compared with the placebo group [2.5 mg losmapimod, −6.7, 95 % CI (−18.2, 4.9), *p* = 0.26; 7.5 mg losmapimod, −4.7, 95 % CI (−16.1, 6.8), *p* = 0.42; and 10 mg losmapimod, −3.4, 95 % CI (−15.1, 8.2), *p* = 0.56].

## Discussion

This systematic review and meta-analysis reviewed existing evidence and concluded the safety and efficacy of p38 MAPKI on the COPD population. Compared with the placebo group, all included p38 MAPKI drugs were safe in adverse events and all concerned systems. However, despite post-bronchodilator FVC in the lung function, no statistically significant efficacy in improving the quality of life, physical endurance, or suppressing inflammation in patients with COPD was observed.

p38 MAPK is a key player in a variety of cellular activities such as inflammation, apoptosis, and proliferation (Ono and Han, 2000). Therefore, side effects that may occur when medicating p38 MAPKI were of great concern (Chopra et al., 2008). The first concern is an infection, which is a general consideration when administrating any medicine with immunosuppressive potency (van den Blink et al., 2001). Second, in the neurological system, p38 MAPKI was considered to be accompanied by potential neurological toxicity for its unexplainably high expression in specific brain areas (Maruyama et al., 2000). Third, cardiotoxicity was focused on p38 MAPKI administration since p38 MAPK in the heart was discovered to be an inhibitor of hypertrophy and a promoter of development in heart tissue (Ma et al., 1999; Ravingerová et al., 2003). Fourth, digestive side-effects including hepatic or gastrointestinal toxicity are theoretically possible and observed in the animal study (Khorasanizadeh et al., 2017). Fifth, other classified or unclassified adverse events, such as skin symptoms, remain unknown. In our study, the assessment related to safety indicates that utilizing p38 MAPKI on patients with COPD might be safe. When compared to placebo, there was no increase in the total incidence of adverse events, exacerbation, or any other adverse events.

P38 MAPKIs was viewed as a promising alternative to corticosteroids due to its anti-inflammatory properties. Activated p38 overexpression was observed in multiple types of cells collected from COPD patients, including alveolar macrophages, alveolar CD8^+^ T cells, and airway epithelial cells, all of which play important roles in the inflammatory response (Renda et al., 2008; Gaffey et al., 2013). Furthermore, data from several studies have shown a close relationship between p38 up-regulation and the level of inflammatory biomarkers (Huang et al., 2013; Vallese et al., 2015). Moreover, FEV1 was found remarkably correlated with the p38 MAPK expression, which indicates that p38 MAPKI may have a direct effect on improving the lung function in COPD population (Huang et al., 2013). However, despite improvement found in FEV1 and FVC, none of the aforementioned potential efficacies was confirmed in our study.

The safety and doubtful efficiency of p38 MAPKI we observed may have the same reasons: 1) p38 MAPK has potential reciprocal redundant agents in cell-signaling cascades, efficacy coming from inhibition of its expression might be compensated through other pathways (Pelaia et al., 2020); 2) Since all of the included subjects were stable patients, the aforementioned compensation might be more robust, and differences might be detected in the case of an exacerbation (Vallese et al., 2015); 3) p38 MAPKI was found to be able to weaken or diminish the resistance of corticosteroids, which indicates that maybe the effect can be presented only when administrating the combined regimen of p38 MAPKI and corticosteroids (Armstrong et al., 2011; Khorasani et al., 2015; Lea et al., 2020).

Though existing pieces of evidence are all extracted from high-quality RCTs, some limitations should be noticed to interpret the results cautiously. First, the number of included studies is only 8, and in which 5 different p38 MAPKIs were reported, potential heterogeneity might impair the strength of the evidence. Second, there was no evidence found comparing the safety and efficacy of different kinds of p38 MAPKIs. Third, only two studies focused on inhaled p38 MAPKIs, which were considered to be the optimal way to administer this type of drug (Millan, 2011). Fourth, hepatic toxicity was discussed insufficiently in all the included studies with many worries about this domain (Pelaia et al., 2020). Fifth, all included studies focused on patients with stable COPD, p38 MAPKIs’ anti-inflammatory efficacy on the exacerbation status of COPD seemed to be a blind spot in the study of such drugs. Sixth, the majority of patients included in the study were European; research on other populations is essentially non-existent. Seventh, some p38 MAPK studies might be discontinued due to safety issues but have not been published, the statements about safety here are only for the drugs included in this study. Eighth, publication bias was not conducted due to the small number of included studies.

As we know, this is the first evidence-based medical study on the safety and efficacy of p38 MAPKI in COPD population. In this study, we systematically reviewed existing RCTs and meta-analyzed the safety and therapeutic value of p38 MAPKI. We have concluded a perspective that p38 MAPKI monotherapy might be safe but ineffective in COPD population based on existing conflicting pieces of evidence. Given the aforementioned shortcomings and the fact that the evidence is still not perfect, future RCTs with larger samples, or head-to-head, or in combination with corticosteroids are still warranted.

## Conclusion

Based on the evidence we gathered, compared with placebo, administrating p38 MAPKIs in patients with COPD may cause neither more adverse events, nor observable efficacy. Its slight improvement on post-bronchodilator FVC remains doubtful. These results should be interpreted with caution since the number of included studies is limited, and heterogeneity from combining different p38 MAPKIs as a whole is unavoidable.

## Data Availability

The original contributions presented in the study are included in the article/Supplementary Materials; further inquiries can be directed to the corresponding author.

## Glossary

AE: adverse events
ANY: any adverse events
CC-16: Clara cell secretory protein 16
CI: confidence interval
CNKI: China National Knowledge Infrastructure
COPD: chronic obstructive pulmonary disease
CRP: c-reactive protein
CV: cardiovascular symptoms
DENT: dental symptoms or symptoms occurred in ear, nose, or throat
ECOPD: exacerbations of chronic obstructive pulmonary disease
FEM: fixed effect model
FEV1: forced expiratory volume in first second
FIB: fibrinogen
FVC: forced vital capacity
hsCRP: high sensitive C-reactive protein;
IB: inflammatory biomarkers
IC: inspiratory capacity
ICTRP: international clinical trials registry platform
IL: interleukin
MMP-9: matrix metalloprotein 9
MP: musculoskeletal pain
NF-kB: nuclear factor kappa B
NS: symptoms of nerve system
p38 MAPK: p38 mitogen-activated protein kinase
p38 MAPKI: p38 mitogen-activated protein kinase inhibitor
PRISMA: Preferred Reporting Items for Systematic Review and Meta-analyses
QoL: quality of life
RCT: randomized controlled trial
REM: random effects model
RI: respiratory infections despite of ECOPD
ROB: risk of bias
RR: risk ratio
RV: residual volume
SAE: severe adverse events
SD: standard difference
SGRQ: St. George Respiratory Questionnaire
SMD: standardized mean difference
SP-D: surfactant protein D
TLC: Total lung capacity
UI: urinary infections
WOS: web of science.

## References

[B1] Armstrong J. Harbron C. Lea S. Booth G. Cadden P. Wreggett K. A. (2011). Synergistic effects of p38 mitogen-activated protein kinase inhibition with a corticosteroid in alveolar macrophages from patients with chronic obstructive pulmonary disease. J. Pharmacol. Exp. Ther. 338 (3), 732–740. 10.1124/jpet.111.180737 21610141

[B2] Banerjee A. Koziol-White C. Panettieri R. Jr (2012). p38 MAPK inhibitors, IKK2 inhibitors, and TNFα inhibitors in COPD. Curr. Opin. Pharmacol. 12 (3), 287–292. 10.1016/j.coph.2012.01.016 22365729 PMC4030417

[B3] Barnes P. J. (2013). Corticosteroid resistance in patients with asthma and chronic obstructive pulmonary disease. J. Allergy Clin. Immunol. 131 (3), 636–645. 10.1016/j.jaci.2012.12.1564 23360759

[B4] Charron C. E. Russell P. Ito K. Lea S. Kizawa Y. Brindley C. (2017). RV568, a narrow-spectrum kinase inhibitor with p38 MAPK-α and -γ selectivity, suppresses COPD inflammation. Eur. Respir. J. 50 (4), 1700188. 10.1183/13993003.00188-2017 29074542

[B5] Chopra P. Kanoje V. Semwal A. Ray A. (2008). Therapeutic potential of inhaled p38 mitogen-activated protein kinase inhibitors for inflammatory pulmonary diseases. Expert Opin. Investig. Drugs 17 (10), 1411–1425. 10.1517/13543784.17.10.1411 18808304

[B6] Chung K. F. (2011). p38 mitogen-activated protein kinase pathways in asthma and COPD. Chest 139 (6), 1470–1479. 10.1378/chest.10-1914 21652557

[B7] Duan W. Chan J. H. McKay K. Crosby J. R. Choo H. H. Leung B. P. (2005). Inhaled p38alpha mitogen-activated protein kinase antisense oligonucleotide attenuates asthma in mice. Am. J. Respir. Crit. Care Med. 171 (6), 571–578. 10.1164/rccm.200408-1006OC 15557129

[B8] Egger M. Davey Smith G. Schneider M. Minder C. (1997). Bias in meta-analysis detected by a simple, graphical test. Bmj 315 (7109), 629–634. 10.1136/bmj.315.7109.629 9310563 PMC2127453

[B9] Escott K. J. Belvisi M. G. Birrell M. A. Webber S. E. Foster M. L. Sargent C. A. (2000). Effect of the p38 kinase inhibitor, SB 203580, on allergic airway inflammation in the rat. Br. J. Pharmacol. 131 (2), 173–176. 10.1038/sj.bjp.0703605 10991908 PMC1572335

[B10] Fisk M. Cheriyan J. Mohan D. Forman J. Mäki-Petäjä K. M. McEniery C. M. (2018). The p38 mitogen activated protein kinase inhibitor losmapimod in chronic obstructive pulmonary disease patients with systemic inflammation, stratified by fibrinogen: A randomised double-blind placebo-controlled trial. PloS one 13 (3), e0194197. 10.1371/journal.pone.0194197 29566026 PMC5863984

[B11] Gaffey K. Reynolds S. Plumb J. Kaur M. Singh D. (2013). Increased phosphorylated p38 mitogen-activated protein kinase in COPD lungs. Eur. Respir. J. 42 (1), 28–41. 10.1183/09031936.00170711 23060629

[B12] Gan W. Q. Man S. F. Senthilselvan A. Sin D. D. (2004). Association between chronic obstructive pulmonary disease and systemic inflammation: A systematic review and a meta-analysis. Thorax 59 (7), 574–580. 10.1136/thx.2003.019588 15223864 PMC1747070

[B13] Garcia-Rio F. Miravitlles M. Soriano J. B. Muñoz L. Duran-Tauleria E. Sánchez G. (2010). Systemic inflammation in chronic obstructive pulmonary disease: A population-based study. Respir. Res. 11 (1), 63. 10.1186/1465-9921-11-63 20500811 PMC2891677

[B14] GBD Chronic Respiratory Disease Collaborators (2020). Prevalence and attributable health burden of chronic respiratory diseases, 1990-2017: A systematic analysis for the global burden of disease study 2017. Lancet. Respir. Med. 8 (6), 585–596. 10.1016/S2213-2600(20)30105-3 32526187 PMC7284317

[B15] Gold Science Committee (2021). Global strategy for prevention, diagnosis and management of COPD: 2022. Report online: https://goldcopd.org/. Available from: https://goldcopd.org/wp-content/uploads/2021/12/GOLD-REPORT-2022-v1.1-22Nov2021_WMV.pdf .

[B16] Hakim A. Adcock I. M. Usmani O. S. (2012). Corticosteroid resistance and novel anti-inflammatory therapies in chronic obstructive pulmonary disease: Current evidence and future direction. Drugs 72 (10), 1299–1312. 10.2165/11634350-000000000-00000 22731962

[B17] Huang C. Xie M. He X. Gao H. (2013). Activity of sputum p38 MAPK is correlated with airway inflammation and reduced FEV1 in COPD patients. Med. Sci. Monit. 19, 1229–1235. 10.12659/MSM.889880 24382347 PMC3890402

[B18] Inc. MUaEP (2022). GRADEpro GDT. Available from: https://gdt.gradepro.org/app/ (Accessed April 30, 2022).

[B19] Khorasani N. Baker J. Johnson M. Chung K. F. Bhavsar P. K. (2015). Reversal of corticosteroid insensitivity by p38 MAPK inhibition in peripheral blood mononuclear cells from COPD. Int. J. Chron. Obstruct. Pulmon. Dis. 10, 283–291. 10.2147/COPD.S72403 25678784 PMC4322842

[B20] Khorasanizadeh M. Eskian M. Gelfand E. W. Rezaei N. (2017). Mitogen-activated protein kinases as therapeutic targets for asthma. Pharmacol. Ther. 174, 112–126. 10.1016/j.pharmthera.2017.02.024 28223227

[B21] Lea S. Li J. Plumb J. Gaffey K. Mason S. Gaskell R. (2020). P38 MAPK and glucocorticoid receptor crosstalk in bronchial epithelial cells. J. Mol. Med. 98 (3), 361–374. 10.1007/s00109-020-01873-3 31974640 PMC7080672

[B22] Lee J. C. Kassis S. Kumar S. Badger A. Adams J. L. (1999). p38 mitogen-activated protein kinase inhibitors--mechanisms and therapeutic potentials. Pharmacol. Ther. 82 (2-3), 389–397. 10.1016/s0163-7258(99)00008-x 10454214

[B23] Lomas D. A. Lipson D. A. Miller B. E. Willits L. Keene O. Barnacle H. (2012). An oral inhibitor of p38 MAP kinase reduces plasma fibrinogen in patients with chronic obstructive pulmonary disease. J. Clin. Pharmacol. 52 (3), 416–424. 10.1177/0091270010397050 22090363

[B24] Ma X. L. Kumar S. Gao F. Louden C. S. Lopez B. L. Christopher T. A. (1999). Inhibition of p38 mitogen-activated protein kinase decreases cardiomyocyte apoptosis and improves cardiac function after myocardial ischemia and reperfusion. Circulation 99 (13), 1685–1691. 10.1161/01.cir.99.13.1685 10190877

[B25] MacNee W. Allan R. J. Jones I. De Salvo M. C. Tan L. F. (2013). Efficacy and safety of the oral p38 inhibitor PH-797804 in chronic obstructive pulmonary disease: A randomised clinical trial. Thorax 68 (8), 738–745. 10.1136/thoraxjnl-2012-202744 23539534

[B26] Marks-Konczalik J. Costa M. Robertson J. McKie E. Yang S. Pascoe S. (2015). A post-hoc subgroup analysis of data from a six month clinical trial comparing the efficacy and safety of losmapimod in moderate-severe COPD patients with ≤2% and >2% blood eosinophils. Respir. Med. 109 (7), 860–869. 10.1016/j.rmed.2015.05.003 26033641

[B27] Maruyama M. Sudo T. Kasuya Y. Shiga T. Hu B. Osada H. (2000). Immunolocalization of p38 MAP kinase in mouse brain. Brain Res. 887 (2), 350–358. 10.1016/s0006-8993(00)03063-8 11134625

[B28] Millan D. S. (2011). What is the potential for inhaled p38 inhibitors in the treatment of chronic obstructive pulmonary disease? Future Med. Chem. 3 (13), 1635–1645. 10.4155/fmc.11.96 21942253

[B29] Norman P. (2015). Investigational p38 inhibitors for the treatment of chronic obstructive pulmonary disease. Expert Opin. Investig. Drugs 24, 383–392. 10.1517/13543784.2015.1006358 25599809

[B30] Ono K. Han J. (2000). The p38 signal transduction pathway: Activation and function. Cell. Signal. 12 (1), 1–13. 10.1016/s0898-6568(99)00071-6 10676842

[B31] Page M. J. McKenzie J. E. Bossuyt P. M. Boutron I. Hoffmann T. C. Mulrow C. D. (2021). The PRISMA 2020 statement: An updated guideline for reporting systematic reviews. Bmj 372, n71. 10.1136/bmj.n71 33782057 PMC8005924

[B32] Pascoe S. Costa M. Marks-Konczalik J. McKie E. Yang S. Scherbovsky P. S. (2017). Biological effects of p38 MAPK inhibitor losmapimod does not translate to clinical benefits in COPD. Respir. Med. 130, 20–26. 10.1016/j.rmed.2017.07.002 29206629

[B33] Patel N. R. Cunoosamy D. M. Fagerås M. Taib Z. Asimus S. Hegelund-Myrbäck T. (2018). The development of AZD7624 for prevention of exacerbations in COPD: A randomized controlled trial. Int. J. Chron. Obstruct. Pulmon. Dis. 13, 1009–1019. 10.2147/COPD.S150576 29628759 PMC5877500

[B34] Pelaia C. Vatrella A. Sciacqua A. Terracciano R. Pelaia G. (2020). Role of p38-mitogen-activated protein kinase in COPD: Pathobiological implications and therapeutic perspectives. Expert Rev. Respir. Med. 14 (5), 485–491. 10.1080/17476348.2020.1732821 32077346

[B35] Pfizer PCgCC (2016). An evaluation of PF-03715455 in moderate to severe chronic obstructive pulmonary disease ClinicalTrials.gov. Available from: https://clinicaltrials.gov/ct2/show/study/NCT02366637?term=NCT02366637&draw=1&rank=1 (Accessed March 15, 2022).

[B36] Ravingerová T. Barancík M. Strnisková M. (2003). Mitogen-activated protein kinases: A new therapeutic target in cardiac pathology. Mol. Cell. Biochem. 247 (1-2), 127–138. 10.1023/a:1024119224033 12841640

[B37] Renda T. Baraldo S. Pelaia G. Bazzan E. Turato G. Papi A. (2008). Increased activation of p38 MAPK in COPD. Eur. Respir. J. 31 (1), 62–69. 10.1183/09031936.00036707 17959643

[B38] Singh D. Smyth L. Borrill Z. Sweeney L. Tal-Singer R. (2010). A randomized, placebo-controlled study of the effects of the p38 MAPK inhibitor SB-681323 on blood biomarkers of inflammation in COPD patients. J. Clin. Pharmacol. 50 (1), 94–100. 10.1177/0091270009347873 19880675

[B39] StatsToDo (2020). StatsToDo : Home page. Available from: https://www.statstodo.com (Accessed March 27, 2022).

[B40] Strâmbu I. R. Kobalava Z. D. Magnusson B. P. MacKinnon A. Parkin J. M. (2019). Phase II study of single/repeated doses of acumapimod (BCT197) to treat acute exacerbations of COPD. Copd 16 (5-6), 344–353. 10.1080/15412555.2019.1682535 31682162

[B41] Vallese D. Ricciardolo F. L. Gnemmi I. Casolari P. Brun P. Sorbello V. (2015). Phospho-p38 MAPK expression in COPD patients and asthmatics and in challenged bronchial epithelium. Respiration. 89 (4), 329–342. 10.1159/000375168 25791156

[B42] van den Blink B. Juffermans N. P. ten Hove T. Schultz M. J. van Deventer S. J. van der Poll T. (2001). p38 mitogen-activated protein kinase inhibition increases cytokine release by macrophages *in vitro* and during infection *in vivo* . J. Immunol. 166 (1), 582–587. 10.4049/jimmunol.166.1.582 11123340

[B43] Watz H. Barnacle H. Hartley B. F. Chan R. (2014). Efficacy and safety of the p38 MAPK inhibitor losmapimod for patients with chronic obstructive pulmonary disease: A randomised, double-blind, placebo-controlled trial. Lancet. Respir. Med. 2 (1), 63–72. 10.1016/S2213-2600(13)70200-5 24461903

[B44] Yu H. Su X. Lei T. (2022). Effect and safety of p38-MAPK inhibitor on patients with COPD: A systematic review and meta-analysis. PROSPERO International prospective register of systematic reviews. Available from: https://www.crd.york.ac.uk/PROSPERO/display_record.php?RecordID=302890 .

