# Supplementary material for: Safety and efficacy of p38 mitogen-activated protein kinase inhibitors (MAPKIs) in COPD
Source: Front Pharmacol. 2022 Sep 28;13:950035. doi: 10.3389/fphar.2022.950035 (PMC9554617; doi:10.3389/fphar.2022.950035)
Supplement: Supplementary file 1 [file DataSheet1.PDF]

Safety and Efficacy of p38 MAPKI in COPD: A Systematic Review.

Supplementary Material Collection

frontiers

CONTENT

Section I. Brief description of statistical analysis 3
Data extraction details: 3
Meta-analysis details....3
Section II. Literature searching process 4
Literature search in PubMed....4
Literature search in Embase....5
Literature search in Cochrane Library 5
Literature search in Web Of Science....6
Literature search in CNKI....8
Literature search in SinoMed....8
Literature search in ICTRP 8
Literature search in ClinicalTrials.gov....8
Screening of the registries retrieved from ClinicalTrials.gov....9
Section III. Full assessment of risk of bias....11
Charron 2017 11
Fisk 2018....12
Lomas 2011....13
MacNee 2013....14
NCT02366637....15
Pascoe 2017 16
Patel 2018....18
Singh 2010 19
Strâmbu 2019....20
Watz 2014 21
Section IV. Results of meta-analysis....22
Safety assessment....22
Any adverse events 22
Severe adverse events 23
Exacerbation of COPD 24
Neurological adverse events 25
Dental adverse events or adverse events occurred in ear, nose or throat. 26
Other respiratory infection....27
Cardiovascular adverse events....28
Digestive adverse events....29
Urinary infection....30
Musculoskeletal pain 31
Efficacy assessment 32
FEV1 pre bronchodilator 32
FEV1 post bronchodilator....33
FVC pre bronchodilator....34
FVC post bronchodilator....35

FEV1/FVC 36
TLC 37
IC 38
SGRQ 39
hsCRP 40
hsCRPratio 41
FIB 42
Section V. GRADE SOF table 43
Table 1: GRADE SOF of p38 MAPKIs' safety in COPD. 43
Table 2: GRADE SOF of p38 MAPKIs' efficacy on COPD. 46
Section VI. PRISMA 2020 Checklist 50

Section I. Brief description of statistical analysis

Data extraction details:

1. When data of different groups were needed to be combined in one, an online tool was used to perform this procedure.
   https://www.statstodo.com/CombineMeansSDs.php
2. When data were presented in the style of "median" and its relating quartiles, an online tool was used to transformed them into "mean" style.
   https://www.math.hkbu.edu.hk/~tongt/papers/median2mean.html
3. When data were presented in the style of figure, Engauge Digitizer (version 11.1) was used to extract them.
4. When data were performed in the style of "mean±SE (in-group)", their SD will be calculated by "SD = SE $\times \sqrt{n}$".
5. All outcomes are extracted and processed by the principle of "change from baseline".
6. When the report provides MD (mean difference) and its 95%CI (confidence interval), we will calculate arm-based data using Review Manager's calculator(manually setting the mean of control groups to "0").

Meta-analysis details

1. All meta-analysis was conducted in R (version 4.1.1)
2. Package "xlsx" (version 0.6.0) was used to induce extracted data into R.
   - Code sample:
     sampledata <- read.xlsx("sample.xlsx", sheetIndex = 1, header = TRUE)
3. Package "meta" (version 5.2-0) was used to calculate all pooling estimation in this study.
   - Code sample
     a) for continuous data:
        sampleresult <- metacont(data = sampledata, n.e, mean.e, sd.e, n.c, mean.c, sd.c, sm = "SMD", studlab = study)
     b) for binary data:
        sampleresult <- metabin(data = sampledata, n.e, total.e, n.c, total.c, sm = "RR", studylab = study)
     c) for sensitivity analysis:
        sampleinf <- metainf(sampleresult, pooled = "random")
4. In R, we could not export the forest plot with the *p* values of pooling estimations, so we manually recorded them.

Section II. Literature searching process
Literature search in PubMed

No.	Query	Results
#5	(#1 OR #3) AND (#2 OR #4)	286
#4	"MAP kinase p38"[Title/Abstract] OR "MAPK p38"[Title/Abstract] OR "Mitogen Activated Protein Kinase p38"[Title/Abstract] OR "Mitogen-Activated Protein Kinase p38"[Title/Abstract] OR "p38 MAP Kinase"[Title/Abstract] OR "p38 MAPK"[Title/Abstract] OR "p38 Mitogen Activated Protein Kinase"[Title/Abstract] OR "p38 Mitogen Activated Protein Kinases"[Title/Abstract] OR "p38 Mitogen-Activated Protein Kinase"[Title/Abstract] OR "p38 Protein Kinase"[Title/Abstract] OR "p38 SAPK"[Title/Abstract] OR "Protein Kinase, p38"[Title/Abstract] OR "protein SAPK2"[Title/Abstract] OR "SAPK2"[Title/Abstract] OR "SAPK2 protein"[Title/Abstract] OR "stress activated protein kinase 2"[Title/Abstract]	26317
#3	"Pulmonary Disease, Chronic Obstructive"[Title/Abstract] OR "chronic obstructive lung disease"[Title/Abstract] OR "Airflow Obstruction, Chronic"[Title/Abstract] OR "Airflow Obstructions, Chronic"[Title/Abstract] OR "Chronic Airflow Obstruction"[Title/Abstract] OR "Chronic Airflow Obstructions"[Title/Abstract] OR "chronic airway obstruction"[Title/Abstract] OR "Chronic Obstructive Airway Disease"[Title/Abstract] OR "Chronic Obstructive Pulmonary Disease"[Title/Abstract] OR "Chronic Obstructive Pulmonary Diseases"[Title/Abstract] OR "chronic obstructive bronchopulmonary disease"[Title/Abstract] OR "chronic obstructive lung disorder"[Title/Abstract] OR "chronic obstructive pulmonary disorder"[Title/Abstract] OR "chronic obstructive respiratory disease"[Title/Abstract] OR "chronic pulmonary obstructive disease"[Title/Abstract] OR "chronic pulmonary obstructive disorder"[Title/Abstract] OR "COAD"[Title/Abstract] OR "COPD"[Title/Abstract] OR "lung chronic obstructive disease"[Title/Abstract] OR "lung disease, chronic obstructive"[Title/Abstract] OR "obstructive chronic lung disease"[Title/Abstract] OR "obstructive chronic pulmonary disease"[Title/Abstract] OR "obstructive lung disease, chronic"[Title/Abstract] OR "pulmonary disorder, chronic obstructive"[Title/Abstract]	76577
#2	"p38 Mitogen-Activated Protein Kinases"[Mesh]	24027
#1	Pulmonary Disease, Chronic Obstructive"[Mesh]	61877

Literature search in Embase

No.	Query	Results
#5	(#1 OR #3) AND (#2 OR #4)	556
#4	'mitogen activated protein kinase p38'/exp	38696
#3	'chronic obstructive lung disease'/exp	154303
#2	'map kinase p38':ti,ab,kw OR 'mapk p38':ti,ab,kw OR 'mitogen activated protein kinase p38':ti,ab,kw OR 'mitogen-activated protein kinase p38':ti,ab,kw OR 'p38 map kinase':ti,ab,kw OR 'p38 mapk':ti,ab,kw OR 'p38 mitogen activated protein kinase':ti,ab,kw OR 'p38 mitogen activated protein kinases':ti,ab,kw OR 'p38 mitogen-activated protein kinase':ti,ab,kw OR 'p38 protein kinase':ti,ab,kw OR 'p38 sapk':ti,ab,kw OR 'protein kinase, p38':ti,ab,kw OR 'protein sapk2':ti,ab,kw OR 'sapk2':ti,ab,kw OR 'sapk2 protein':ti,ab,kw OR 'stress activated protein kinase 2':ti,ab,kw	32023
#1	'pulmonary disease, chronic obstructive':ti,ab,kw OR 'chronic obstructive lung disease':ti,ab,kw OR 'airflow obstruction, chronic':ti,ab,kw OR 'airflow obstructions, chronic':ti,ab,kw OR 'chronic airflow obstruction':ti,ab,kw OR 'chronic airflow obstructions':ti,ab,kw OR 'chronic airway obstruction':ti,ab,kw OR 'chronic obstructive airway disease':ti,ab,kw OR 'chronic obstructive pulmonary disease':ti,ab,kw OR 'chronic obstructive pulmonary diseases':ti,ab,kw OR 'chronic obstructive bronchopulmonary disease':ti,ab,kw OR 'chronic obstructive lung disorder':ti,ab,kw OR 'chronic obstructive pulmonary disorder':ti,ab,kw OR 'chronic obstructive respiratory disease':ti,ab,kw OR 'chronic pulmonary obstructive disease':ti,ab,kw OR 'chronic pulmonary obstructive disorder':ti,ab,kw OR 'coad':ti,ab,kw OR 'copd':ti,ab,kw OR 'lung chronic obstructive disease':ti,ab,kw OR 'lung disease, chronic obstructive':ti,ab,kw OR 'obstructive chronic lung disease':ti,ab,kw OR 'obstructive chronic pulmonary disease':ti,ab,kw OR 'obstructive lung disease, chronic':ti,ab,kw OR 'pulmonary disorder, chronic obstructive':ti,ab,kw	133031

Literature search in Cochrane Library

No.	Query	Results
#5	(#1 OR #3) AND (#2 OR #4)	29

#4	MeSH descriptor: [p38 Mitogen-Activated Protein Kinases] explode all trees	78
#3	MeSH descriptor: [Pulmonary Disease, Chronic Obstructive] explode all trees	6174
#2	(‘MAP kinase p38’ OR ‘MAPK p38’ OR ‘Mitogen Activated Protein Kinase p38’ OR ‘Mitogen-Activated Protein Kinase p38’ OR ‘p38 MAP Kinase’ OR ‘p38 MAPK’ OR ‘p38 Mitogen Activated Protein Kinase’ OR ‘p38 Mitogen Activated Protein Kinases’ OR ‘p38 Mitogen-Activated Protein Kinase’ OR ‘p38 Protein Kinase’ OR ‘p38 SAPK’ OR ‘Protein Kinase, p38’ OR ‘protein SAPK2’ OR ‘SAPK2’ OR ‘SAPK2 protein’ OR ‘stress activated protein kinase 2’):ti,ab,kw	284
#1	(‘Pulmonary Disease, Chronic Obstructive’ OR ‘chronic obstructive lung disease’ OR ‘Airflow Obstruction, Chronic’ OR ‘Airflow Obstructions, Chronic’ OR ‘Chronic Airflow Obstruction’ OR ‘Chronic Airflow Obstructions’ OR ‘chronic airway obstruction’ OR ‘Chronic Obstructive Airway Disease’ OR ‘Chronic Obstructive Pulmonary Disease’ OR ‘Chronic Obstructive Pulmonary Diseases’ OR ‘chronic obstructive bronchopulmonary disease’ OR ‘chronic obstructive lung disorder’ OR ‘chronic obstructive pulmonary disorder’ OR ‘chronic obstructive respiratory disease’ OR ‘chronic pulmonary obstructive disease’ OR ‘chronic pulmonary obstructive disorder’ OR ‘COAD’ OR ‘COPD’ OR ‘lung chronic obstructive disease’ OR ‘lung disease, chronic obstructive’ OR ‘obstructive chronic lung disease’ OR ‘obstructive chronic pulmonary disease’ OR ‘obstructive lung disease, chronic’ OR ‘pulmonary disorder, chronic obstructive’):ti,ab,kw	22788

Literature search in Web Of Science

No.	Query	Results
#4	#1 AND #2 AND #3	78
#3	AB=((random) OR (controll) OR (trial))	2189232
#2	TI=((Pulmonary Disease, Chronic Obstructive) OR (chronic obstructive lung disease) OR (Airflow Obstruction, Chronic) OR (Airflow Obstructions, Chronic) OR (Chronic Airflow Obstruction) OR (Chronic Airflow Obstructions) OR (chronic airway obstruction) OR (Chronic Obstructive Airway Disease) OR (Chronic Obstructive Pulmonary Disease) OR (Chronic Obstructive Pulmonary Diseases) OR (chronic obstructive	574850

	bronchopulmonary disease) OR (chronic obstructive lung disorder) OR (chronic obstructive pulmonary disorder) OR (chronic obstructive respiratory disease) OR (chronic pulmonary obstructive disease) OR (chronic pulmonary obstructive disorder) OR (cold) OR (COPD) OR (lung chronic obstructive disease) OR (lung disease, chronic obstructive) OR (obstructive chronic lung disease) OR (obstructive chronic pulmonary disease) OR (obstructive lung disease, chronic) OR (pulmonary disorder, chronic obstructive)) OR AB=((Pulmonary Disease, Chronic Obstructive) OR (chronic obstructive lung disease) OR (Airflow Obstruction, Chronic) OR (Airflow Obstructions, Chronic) OR (Chronic Airflow Obstruction) OR (Chronic Airflow Obstructions) OR (chronic airway obstruction) OR (Chronic Obstructive Airway Disease) OR (Chronic Obstructive Pulmonary Disease) OR (Chronic Obstructive Pulmonary Diseases) OR (chronic obstructive bronchopulmonary disease) OR (chronic obstructive lung disorder) OR (chronic obstructive pulmonary disorder) OR (chronic obstructive respiratory disease) OR (chronic pulmonary obstructive disease) OR (chronic pulmonary obstructive disorder) OR (cold) OR (COPD) OR (lung chronic obstructive disease) OR (lung disease, chronic obstructive) OR (obstructive chronic lung disease) OR (obstructive chronic pulmonary disease) OR (obstructive lung disease, chronic) OR (pulmonary disorder, chronic obstructive)) OR AK=((Pulmonary Disease, Chronic Obstructive) OR (chronic obstructive lung disease) OR (Airflow Obstruction, Chronic) OR (Airflow Obstructions, Chronic) OR (Chronic Airflow Obstruction) OR (Chronic Airflow Obstructions) OR (chronic airway obstruction) OR (Chronic Obstructive Airway Disease) OR (Chronic Obstructive Pulmonary Disease) OR (Chronic Obstructive Pulmonary Diseases) OR (chronic obstructive bronchopulmonary disease) OR (chronic obstructive lung disorder) OR (chronic obstructive pulmonary disorder) OR (chronic obstructive respiratory disease) OR (chronic pulmonary obstructive disease) OR (chronic pulmonary obstructive disorder) OR (cold) OR (COPD) OR (lung chronic obstructive disease) OR (lung disease, chronic obstructive) OR (obstructive chronic lung disease) OR (obstructive chronic pulmonary disease) OR (obstructive lung disease, chronic) OR (pulmonary disorder, chronic obstructive))	
#1	TI=((MAP kinase p38) OR (MAPK p38) OR (Mitogen Activated Protein Kinase p38) OR (Mitogen-Activated Protein Kinase p38)	54,484

	OR (p38 MAP Kinase) OR (p38 MAPK) OR (p38 Mitogen Activated Protein Kinase) OR (p38 Mitogen Activated Protein Kinases) OR (p38 Mitogen-Activated Protein Kinase) OR (p38 Protein Kinase) OR (p38 SAPK) OR (Protein Kinase, p38) OR (protein SAPK2) OR (SAPK2) OR (SAPK2 protein) OR (stress activated protein kinase 2)) OR AB=((MAP kinase p38) OR (MAPK p38) OR (Mitogen Activated Protein Kinase p38) OR (Mitogen-Activated Protein Kinase p38) OR (p38 MAP Kinase) OR (p38 MAPK) OR (p38 Mitogen Activated Protein Kinase) OR (p38 Mitogen Activated Protein Kinases) OR (p38 Mitogen-Activated Protein Kinase) OR (p38 Protein Kinase) OR (p38 SAPK) OR (Protein Kinase, p38) OR (protein SAPK2) OR (SAPK2) OR (SAPK2 protein) OR (stress activated protein kinase 2)) OR AK=((MAP kinase p38) OR (MAPK p38) OR (Mitogen Activated Protein Kinase p38) OR (Mitogen-Activated Protein Kinase p38) OR (p38 MAP Kinase) OR (p38 MAPK) OR (p38 Mitogen Activated Protein Kinase) OR (p38 Mitogen Activated Protein Kinases) OR (p38 Mitogen-Activated Protein Kinase) OR (p38 Protein Kinase) OR (p38 SAPK) OR (Protein Kinase, p38) OR (protein SAPK2) OR (SAPK2) OR (SAPK2 protein) OR (stress activated protein kinase 2))	

Literature search in CNKI

No.	Query	Results
#1	(全文=(p38 MAPK)) AND (篇关摘=(慢性阻塞性肺 + 慢阻肺 + COPD)) AND (篇关摘=(对照))<br>检索范围: 学术期刊	106

Literature search in SinoMed

No.	Query	Results
#1	"p38"[全部字段:智能] AND "MAPK"[全部字段:智能] AND(""慢性阻塞性肺""[标题:智能] OR ""COPD""[标题:智能] OR ""慢阻肺""[标题:智能]) AND ""对照""[全部字段:智能]	19

Literature search in ICTRP

No.	Query	Results
#1	"COPD" AND "p38 MAPK"	0

Literature search in ClinicalTrials.gov

No.	Query	Results
#1	p38 MAPK | COPD	3

#2	p38|COPD	7
#3	mapk|COPD	5

Screening of the registries retrieved from ClinicalTrials.gov

No.	Results #NCT	Condition
#1	#NCT	Condition
	NCT00642148	This report has been already included in our analysis. (Lomas 2011)
	NCT00144859	This study was claimed completed but with no report published.
	NCT01541852	This report has been already included in our analysis. (Fisk 2018)
#2	#NCT	Condition
	NCT02815488	Terminated without results for "very poor recruitment in the Part 4 of the study"
	NCT01561625	Completed but the intervention is unfit for this study.
	NCT00642148	This report has been already included in our analysis. (Lomas 2011)
	NCT00144859	This study was claimed completed but with no report published.
	NCT02366637	Terminated with results posted on the ClinicalTrials.gov, with a statement claiming "Study terminated on 7 April 2015 for business reasons. No safety and/or efficacy concerns contributed to the termination of the study".<br>This study has been added to our analysis.
	NCT01541852	This report has been already included in our analysis. (Fisk 2018)
	NCT00439881	This study was claimed completed but with no report published. The participants are unfit for this study(arthritis).
#3	#NCT	Condition
	NCT02815488	Terminated without results for "very poor recruitment in

		the Part 4 of the study"
	NCT00642148	This report has been already included in our analysis. (Lomas 2011)
	NCT00144859	This study was claimed completed but with no report published.
	NCT01360931	The participants are unfit for this study(lung cancer) and this is not a interventional study.
	NCT01541852	This report has been already included in our analysis. (Fisk 2018)

Section III. Full assessment of risk of bias
Charron 2017

Bias	Judgement	Support
Random sequence generation (selection bias)	Unclear risk	"Subjects were randomised (1:1:1 ratio) to receive RV568 50 µg, RV568 100 µg or placebo once daily for 14 days."<br><br>No detailed description was found in this report.
Allocation concealment (selection bias)	Unclear risk	Not mentioned.
Blinding of participants and personnel (performance bias)	Unclear risk	Not mentioned.
Blinding of outcome assessment (detection bias)	Unclear risk	Not mentioned.
Incomplete outcome data (attrition bias)	Low risk	"Two patients were withdrawn from the study due to a fall in FEV1>20% from the baseline value; one was withdrawn after a single RV568 100 µg dose with no accompanying symptom changes, the other after 6 days treatment with RV568 50 µg with flu-like symptoms. "<br><br>Cases who lost follow-up were described in detail.
Selective reporting (reporting bias)	Low risk	A randomised, double-blind, parallel-group study (clinical trials.gov NCT01475292) was conducted at the Medicines Evaluation Unit (Manchester, UK).
Other bias	Unclear risk	Not known.

Fisk 2018

Bias	Judgement	Support
Random sequence generation (selection bias)	Low risk	"Participants will be randomised to the Losmapimod or Placebo regimen in a 1:1 ratio. The randomisation will be stratified by treatment centre."
Allocation concealment (selection bias)	Low risk	"The placebo tablets will be manufactured to appear identical to the Losmapimod tablets. Packaging and labelling at point of supply to the patient will be blinded against the active preparation. "
Blinding of participants and personnel (performance bias)	Low risk	"All trial personnel, including investigators giving trial interventions, assessing outcomes, and analysis ofdata, and patients were blinded throughout the study to treatment allocation."
Blinding of outcome assessment (detection bias)	Low risk	"All trial personnel, including investigators giving trial interventions, assessing outcomes, and analysis ofdata, and patients were blinded throughout the study to treatment allocation."
Incomplete outcome data (attrition bias)	Low Risk	"The modified intention-to-treat population consisted of 294 randomized subjects who received at least 1 dose of study medication and had at least 1 on-treatment efficacy assessment (Table I)."
Selective reporting (reporting bias)	Low Risk	Study was registered on ClinicalTrial.gov (NCT01541852) and this report basically followed the protocol. ccc
Other bias	Low risk	Not Known but overall this study was well-designed and completely conducted, therefore we think it is of low risk of other kind of potential bias.

Lomas 2011

Bias	Judgement	Support
Random sequence generation (selection bias)	Low risk	"…using a computer-generated permuted block schedule with stratification by country and smoking status (Figure 1)."
Allocation concealment (selection bias)	Low risk	"Patients randomized to losmapimod received a twice-daily placebo inhalation, and patients randomized to SFC received placebo tablets to match losmapimod."
Blinding of participants and personnel (performance bias)	Low risk	See in online registration: "Masking: Quadruple (Participant, Care Provider, Investigator, Outcomes Assessor)"
Blinding of outcome assessment (detection bias)	Low risk	See in online registration: "Masking: Quadruple (Participant, Care Provider, Investigator, Outcomes Assessor)"
Incomplete outcome data (attrition bias)	Low Risk	"The modified intention-to-treat population consisted of 294 randomized subjects who received at least 1 dose of study medication and had at least 1 on-treatment efficacy assessment (Table I)."
Selective reporting (reporting bias)	Low Risk	Study was registered on ClinicalTrial.gov (NCt00642148) and this report basically followed the protocol. ccc
Other bias	Low risk	Not Known but overall this study was well-designed and completely conducted, therefore we think it is of low risk of other kind of potential bias.

MacNee 2013

Bias	Judgement	Support
Random sequence generation (selection bias)	Low risk	"Patients were initially randomised (according to a computer-generated randomisation code) to one of five treatment groups in the ratio 1:1:1:2:1 for placebo, 0.5 mg, 3 mg, 6 mg, 10 mg once daily PH-797804, respectively, until the interim analysis. ."
Allocation concealment (selection bias)	Low risk	"Trial treatments were supplied as PH-797804 capsules and matching placebo. Patients, investigator staff and the sponsor's project team were masked to treatment assignment throughout the trial until the database was locked. "
Blinding of participants and personnel (performance bias)	Low risk	"Trial treatments were supplied as PH-797804 capsules and matching placebo. Patients, investigator staff and the sponsor's project team were masked to treatment assignment throughout the trial until the database was locked. "
Blinding of outcome assessment (detection bias)	Low risk	"Trial treatments were supplied as PH-797804 capsules and matching placebo. Patients, investigator staff and the sponsor's project team were masked to treatment assignment throughout the trial until the database was locked. "
Incomplete outcome data (attrition bias)	Low Risk	See in Figure 1 of this report.
Selective reporting (reporting bias)	Low Risk	Study was registered on ClinicalTrial.gov (NCT00559910) and this report basically followed the protocol. ccc
Other bias	Low risk	Not Known but overall this study was well-designed and completely conducted, therefore we think it is of low risk of other kind of potential bias.

NCT02366637

Bias	Judgement	Support
Random sequence generation (selection bias)	Unclear risk	No detailed description was found in this registration.
Allocation concealment (selection bias)	Unclear risk	No detailed description was found in this registration.
Blinding of participants and personnel (performance bias)	Low risk	Masking: Triple (Participant, Care Provider, Investigator)
Blinding of outcome assessment (detection bias)	Low risk	Masking: Triple (Participant, Care Provider, Investigator)
Incomplete outcome data (attrition bias)	Low risk	At week 4, most participants was still in cohort and we only extract data before this timepoint.
Selective reporting (reporting bias)	Unclear risk	This study was not published.
Other bias	Unclear risk	Not known.

Pascoe 2017

Bias	Judgement	Support
Random sequence generation (selection bias)	Unclear risk	"In this double-blind, parallel-group study, subjects at risk of COPD exacerbations and ≤2% blood eosinophils at screening, were randomized 1:1 to losmapimod 15 mg or placebo (variable treatment duration: 26-52 weeks). "<br><br>No detailed description was found in this report.
Allocation concealment (selection bias)	Unclear	Not mentioned.
Blinding of participants and personnel (performance bias)	Low risk	"Only the GSK statistician was unblinded to the data in order to perform the interim analysis. The statistician remained blinded to all the safety data."
Blinding of outcome assessment (detection bias)	Low risk	"Only the GSK statistician was unblinded to the data in order to perform the interim analysis. The statistician remained blinded to all the safety data."
Incomplete outcome data (attrition bias)	Low risk	"Data for all randomized subjects was included in the final analyses."<br>"Of 365 subjects screened, 94 subjects were randomized to placebo and 90 to losmapimod 15 mg, and 14 and 10 subjects respectively completed the study (Figure 2). The primary reason for withdrawal was due to the early study termination<br>(Placebo: N=66; losmapimod 15 mg: N=55)."
Selective reporting (reporting bias)	Low risk	"This was a phase 2, randomized, double-blind (sponsor unblinded*), parallel-group, multi-centre study evaluating losmapimod 15 mg twice daily versus placebo in addition to standard care (Protocol 201496; NCT02299375). "
Other bias	Unclear risk	Not known.

Patel 2018

Bias	Judgement	Support
Random sequence generation (selection bias)	Unclear risk	No detailed description was found in this report and its supplementary material.
Allocation concealment (selection bias)	Low risk	"After the run-in period, oral steroids were discontinued and the patients were randomly assigned to a 12-week treatment period in a 1:1 ratio to receive AZD7624 (1.0 mg delivered dose) or matching placebo administered once daily via two inhalations using a novel Aqueous Droplet Inhaler (Scandinavian Health AB, Nacka Strand, Sweden). Study schema are available in the Supplementary material (Figure S2)."
Blinding of participants and personnel (performance bias)	Low risk	See in online registration: "Masking: Triple (Participant, Investigator, Outcomes Assessor)."
Blinding of outcome assessment (detection bias)	Low risk	See in online registration: "Masking: Triple (Participant, Investigator, Outcomes Assessor)."
Incomplete outcome data (attrition bias)	Low risk	"This comprises all patients randomized into the study who receive at least 1 inhalation of study drug and will be analyzed according to randomized treatment (intention-to-treat principle)."
Selective reporting (reporting bias)	Low risk	"These trials are registered with ClinicaTrials.gov (ClinicalTrials.gov identifier: NCT01937338 and NCT02238483)."
Wa	Unclear risk	Not Known.

Singh 2010

Bias	Judgement	Support
Random sequence generation (selection bias)	Unclear risk	No detailed description was found in this report and its supplementary material.
Allocation concealment (selection bias)	Unclear risk	No detailed description was found in this report and its supplementary material.
Blinding of participants and personnel (performance bias)	Low risk	"Masking: Double (Participant, Investigator)"
Blinding of outcome assessment (detection bias)	Low risk	"Masking: Double (Participant, Investigator)"
Incomplete outcome data (attrition bias)	Low risk	"Thirteen patients completed the study. Four patients were withdrawn because of adverse events,<br>which are described later in the Results section. "
Selective reporting (reporting bias)	Low risk	Study was registered on ClinicalTrial.gov (NCT00380133) and this report basically followed the protocol.
Other bias	Low risk	Not Known but overall this study was well-designed and completely conducted, therefore we think it is of low risk of other kind of potential bias.

Strâmbu 2019

Bias	Judgement	Support
Random sequence generation (selection bias)	Low risk	"At Day 1, all eligible patients were randomized via the Interactive Response Technology provider to one of the treatment arms. "
Allocation concealment (selection bias)	Low risk	"Placebo was provided in visually identical capsules to the active therapies."
Blinding of participants and personnel (performance bias)	Low risk	"Patients, investigator staff and all personnel performing the assessments remained blinded to the identity of the treatment from the time of randomization until database lock. "
Blinding of outcome assessment (detection bias)	Low risk	"Patients, investigator staff and all personnel performing the assessments remained blinded to the identity of the treatment from the time of randomization until database lock. "
Incomplete outcome data (attrition bias)	Low Risk	"In total, 183 patients were randomized to treatment and 169 patients (92%) completed the study; four withdrew consent, four were lost to follow-up and six died (unrelated to study drug) (Figure 1). While there were protocol deviations in the study, none were major deviations that led to a subject's data being excluded from the analysis. As such, all 183 patients were included in the efficacy and safety analyses. Baseline characteristics were similar among treatment groups (Table 1).
Selective reporting (reporting bias)	Low Risk	Study was registered on ClinicalTrial.gov (NCT01332097) and this report basically followed the protocol.
Other bias	Low risk	Not Known but overall this study was well-designed and completely conducted, therefore we think it is of low risk of other kind of potential bias.

Watz 2014

(Reports of *Marks Konczalik et al.* is a post hoc analysis of the *Watz et al.*)

Bias	Judgement	Support
Random sequence generation (selection bias)	Low risk	"The randomisation schedule was generated with the GlaxoSmithKline randomisation software RandAll. Randomisation was stratifi ed by country and by COPD exacerbations in the past year (≥2 vs <2). "
Allocation concealment (selection bias)	Low risk	"Losmapimod and placebo tablets were identical in appearance."
Blinding of participants and personnel (performance bias)	Low risk	"Investigators giving treatment, assessing outcomes, and analysing data, and patients were masked to treatment assignment."
Blinding of outcome assessment (detection bias)	Low risk	"Investigators giving treatment, assessing outcomes, and analysing data, and patients were masked to treatment assignment."
Incomplete outcome data (attrition bias)	Low risk	"The primary outcome was change in 6 min walking distance between baseline and week 24, assessed in the intention-to-treat population."
Selective reporting (reporting bias)	Low risk	"This study is registered with ClinicalTrial.gov, number NCT01218126."
Other bias	Low risk	Not Known but overall this study was well-designed and completely conducted, therefore we think it is of low risk of other kind of potential bias.

Section IV. Results of meta-analysis

Safety assessment

Any adverse events

1) Forest plot

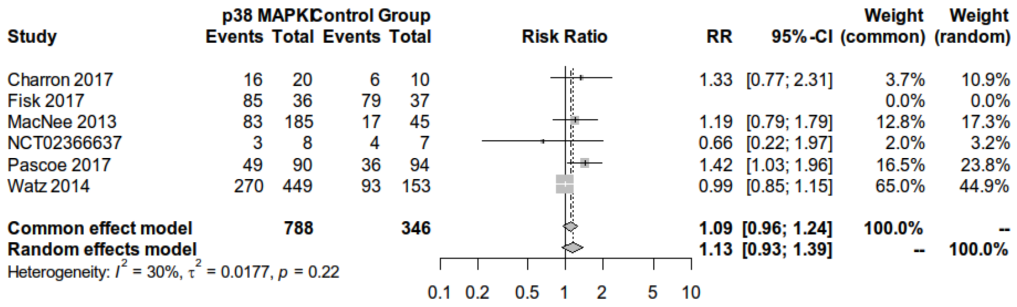

$p = 0.16$(for common effect model)

2) Sensitivity analysis

The I^2 is less than 50%, sensitivity analysis was not conducted for this outcome.

3) Analysis with the exception of *Charron et al.*

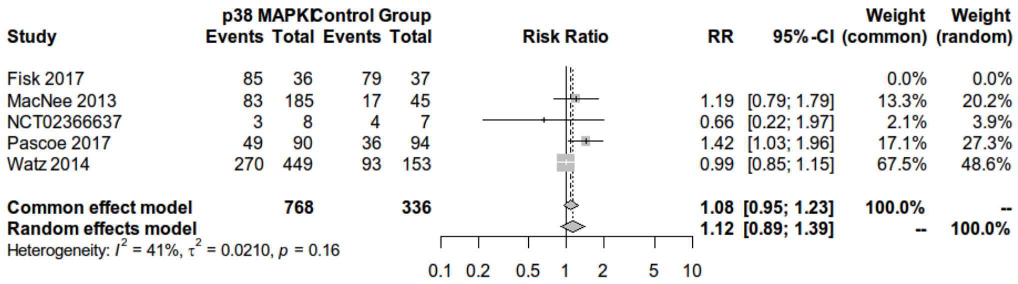

$p = 0.23$(for common effect model)

Severe adverse events

1) Forest plot

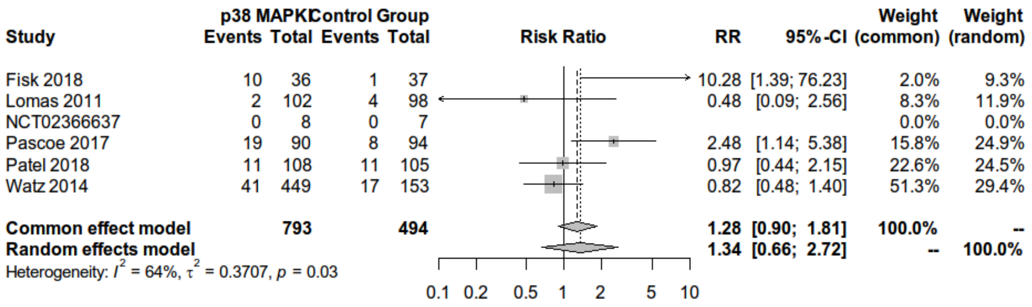

p = 0.42(for random effect model)

2) Sensitivity analysis

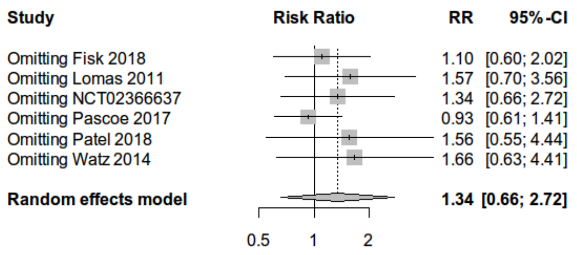

The heterogeneity among included trials did not impair the stability of pooling estimation.

Exacerbation of COPD

1) Forest plot

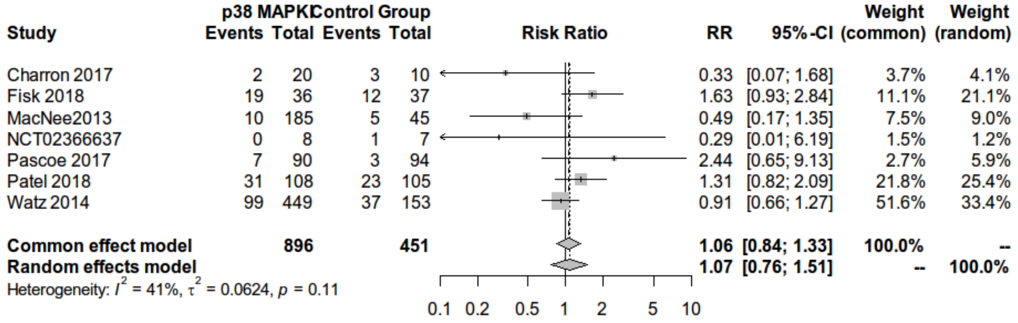

p = 0.63(for random effect model)

2) Sensitivity analysis

The I^2 is less than 50%, sensitivity analysis was not conducted for this outcome.

3) Analysis with the exception of *Charron et al.*

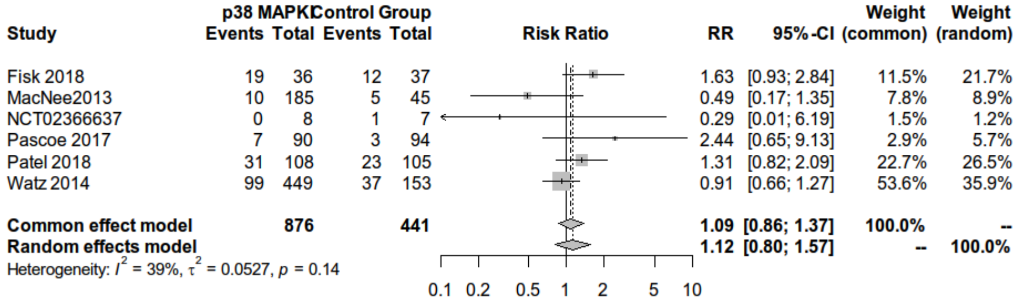

p = 0.49(for common effect model)

Neurological adverse events

1) Forest plot

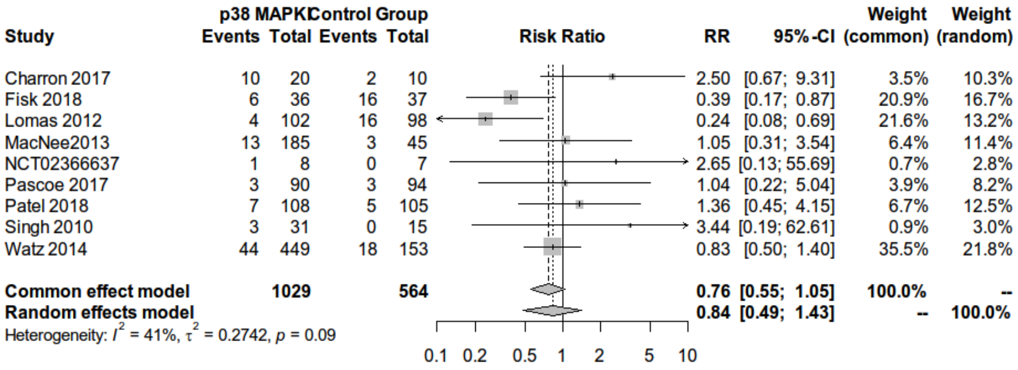

p = 0.10(for common effect model)

2) Sensitivity analysis

The I^2 is less than 50%, sensitivity analysis was not conducted for this outcome.

3) Analysis with the exception of *Charron et al.*

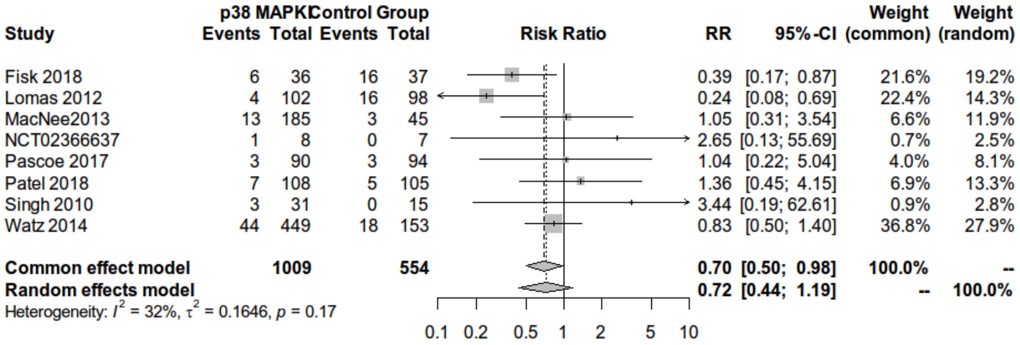

p = 0.04 (for common effect model)

Dental adverse events or adverse events occurred in ear, nose or throat.

1) Forest plot

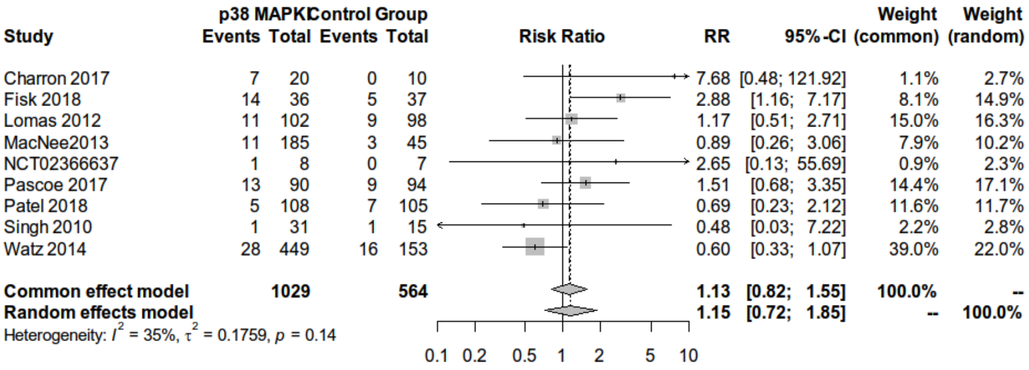

p = 0.47(for common effect model)

2) Sensitivity analysis

The I^2 is less than 50%, sensitivity analysis was not conducted for this outcome.

3) Analysis with the exception of *Charron et al.*

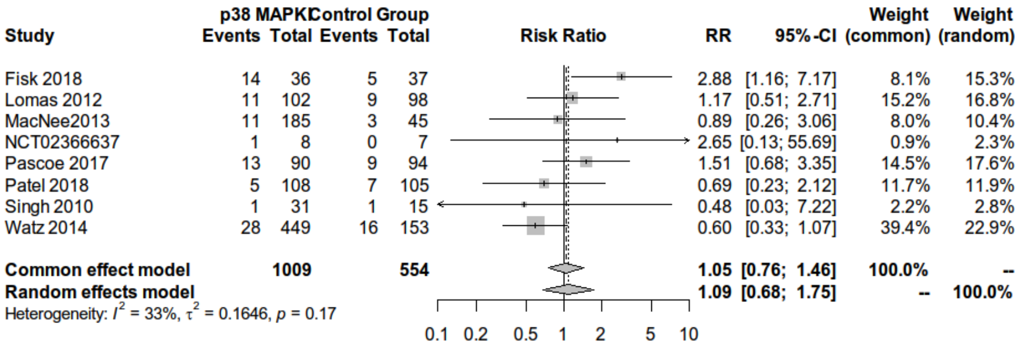

p = 0.76 (for common effect model)

Other respiratory infection

1) Forest plot

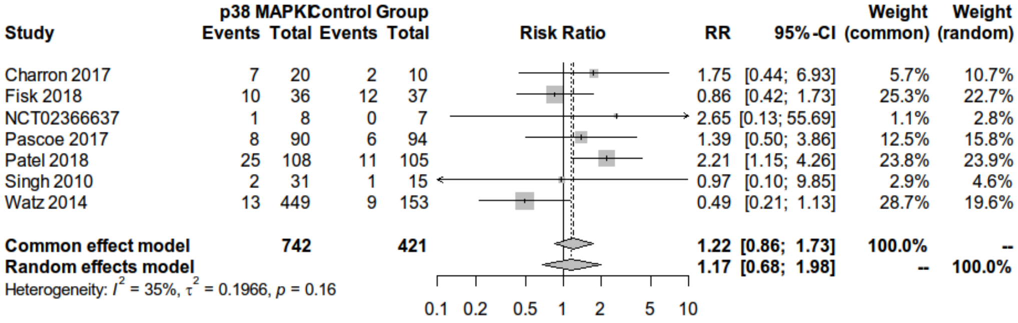

p = 0.27(for common effect model)

2) Sensitivity analysis

The I^2 is less than 50%, sensitivity analysis was not conducted for this outcome.

3) Analysis with exception of the *Charron et al.*

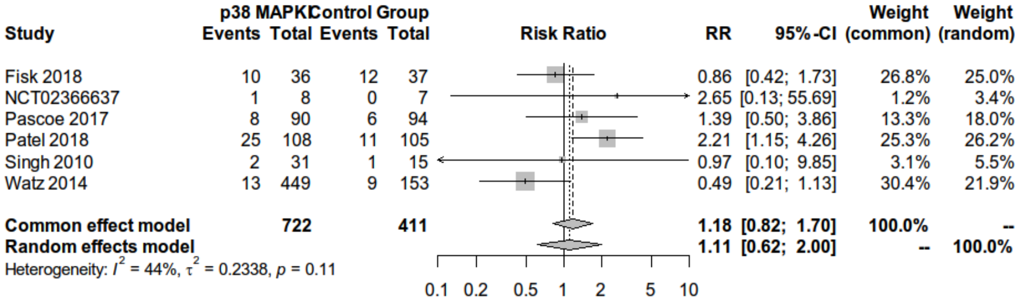

p = 0.36(for common effect model)

Cardiovascular adverse events

1) Forest plot

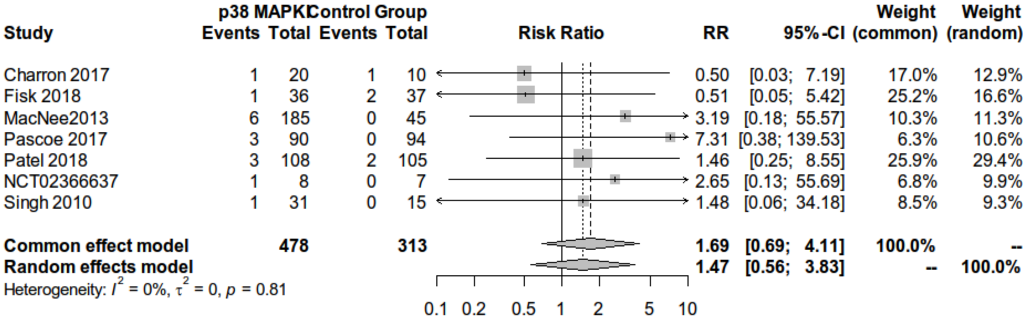

p = 0.25 (for common effect model)

2) Sensitivity analysis

The I^2 is less than 50%, sensitivity analysis was not conducted for this outcome.

3) Analysis with the exception of *Charron et al.*

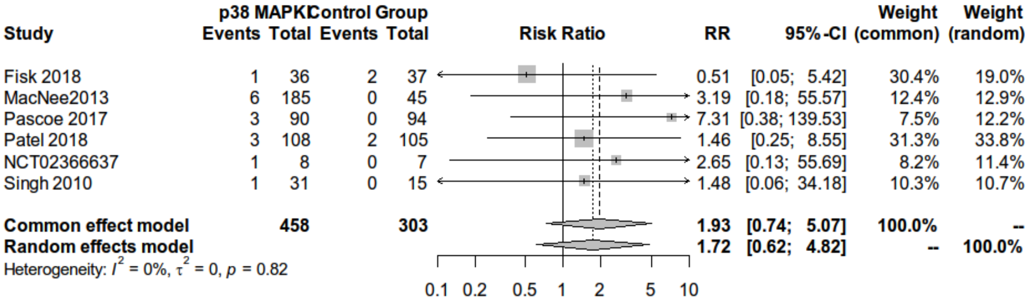

p = 0.18(for common effect model)

Digestive adverse events

1) Forest plot

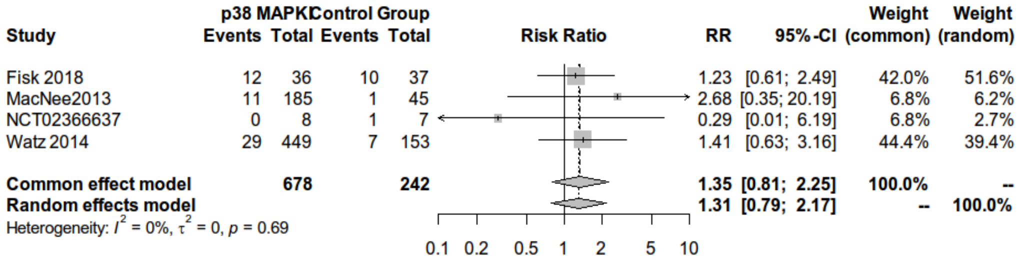

$p = 0.25$ (for common effect model)

2) Sensitivity analysis

The I^2 is less than 50%, sensitivity analysis was not conducted for this outcome.

Urinary infection

1) Forest plot

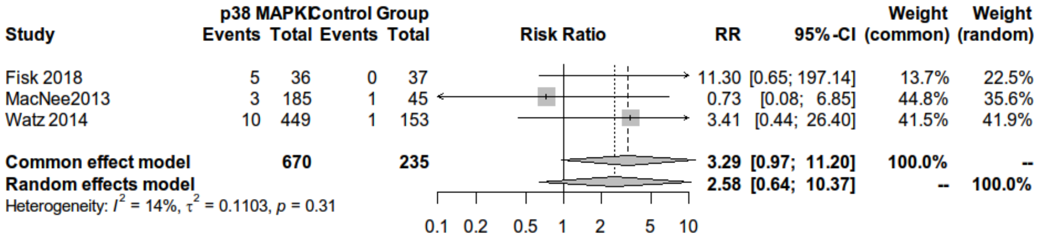

$p = 0.06$(for common effect model)

2) Sensitivity analysis

The I2 is less than 50%, sensitivity analysis was not conducted for this outcome.

Musculoskeletal pain

1) Forest plot

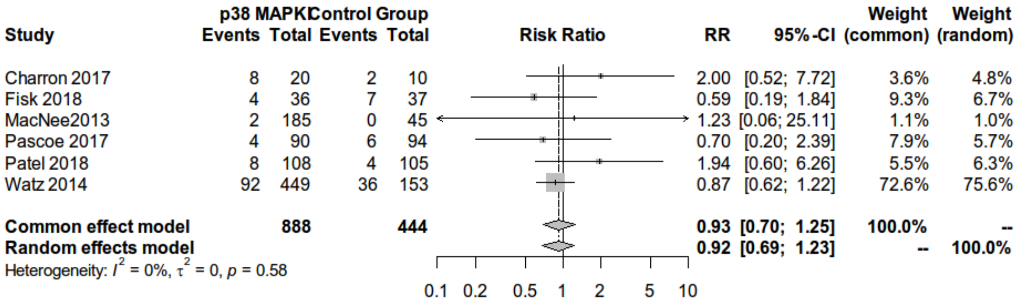

$p = 0.65$(for common effect model)

2) Sensitivity analysis

The I^2 is less than 50%, sensitivity analysis was not conducted for this outcome.

3) Analysis with the exception of *Charron et al.*

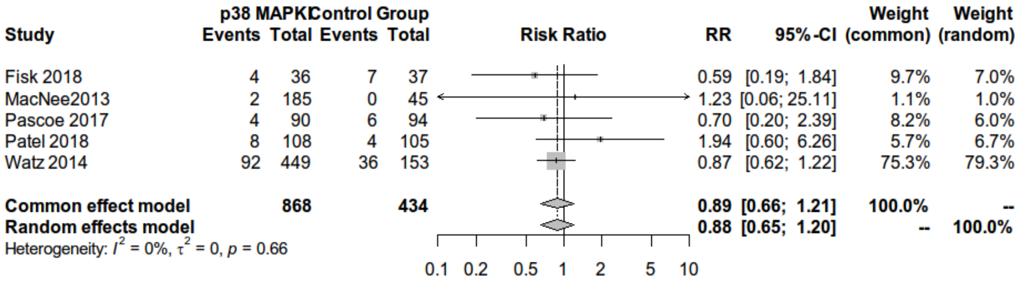

$p = 0.47$(for common effect model)

Efficacy assessment

FEV1 pre bronchodilator

1) Forest plot

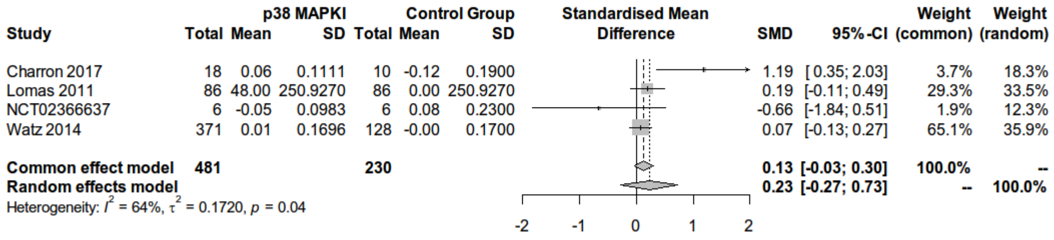

p = 0.38(for random effect model)

2) Sensitivity analysis

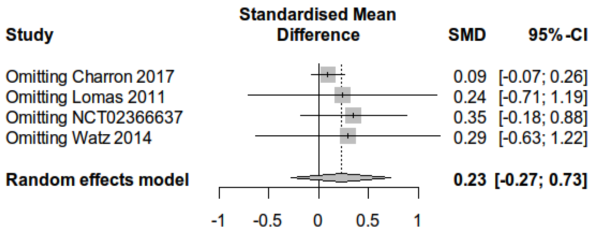

The heterogeneity among included trials does not impair the stability of pooling estimation.

3) Analysis with the exception of *Charron et al.*

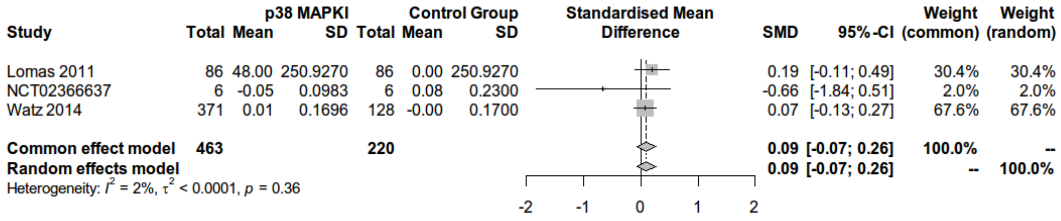

p = 0.27(for common effect model)

FEV1 post bronchodilator

1) Forest analysis

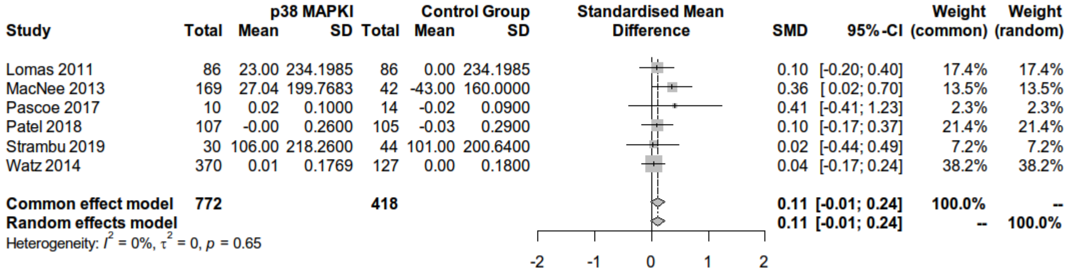

p = 0.08(for common effect model)

2) Sensitivity analysis

The I^2 is less than 50%, sensitivity analysis was not conducted for this outcome.

FVC pre bronchodilator

1) Forest plot

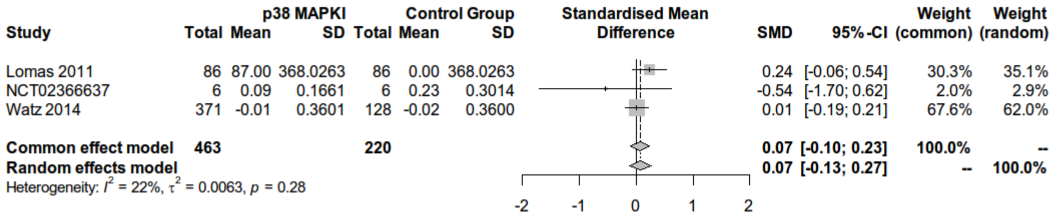

p = 0.43 (for common effect model)

2) Sensitivity analysis

The I^2 is less than 50%, sensitivity analysis was not conducted for this outcome.

FVC post bronchodilator

1) Forest plot

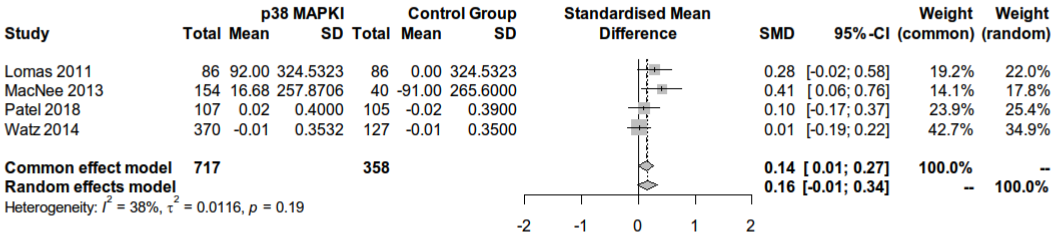

p = 0.03(for common effect model)

2) Sensitivity analysis

The I^2 is less than 50%, sensitivity analysis was not conducted for this outcome.

FEV1/FVC

1) Forest plot

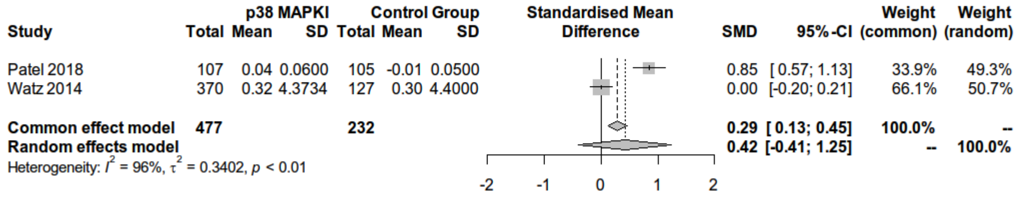

$p = 0.32$(for common effect model)

2) Sensitivity analysis

There is only 2 studies included in this outcome, so there is no need for sensitivity analysis.

TLC

1) Forest plot

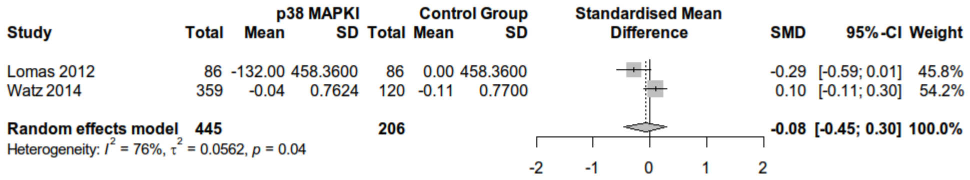

p = 0.68(for random effect model)

2) Sensitivity analysis

There is only 2 studies included in this outcome, so there is no need for sensitivity analysis.

IC

1) Forest plot

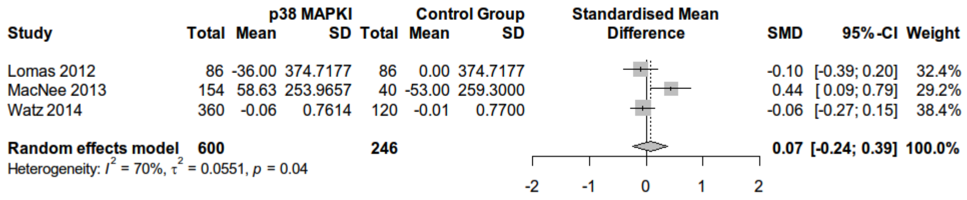

$p = 0.65$(for random effect model)

2) Sensitivity analysis

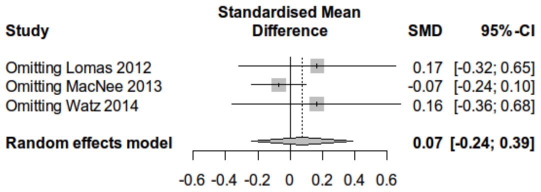

The heterogeneity among included trials does not impair the stability of pooling estimation.

SGRQ

1) Forest plot

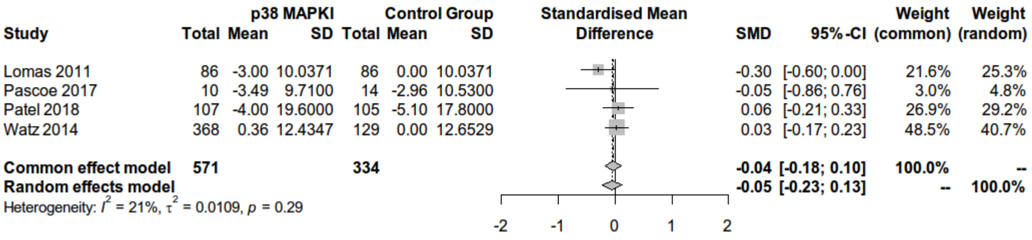

$p = 0.61$(for common effect model)

2) Sensitivity analysis

The I^2 is less than 50%, sensitivity analysis was not conducted for this outcome.

hsCRP

1) Forest plot

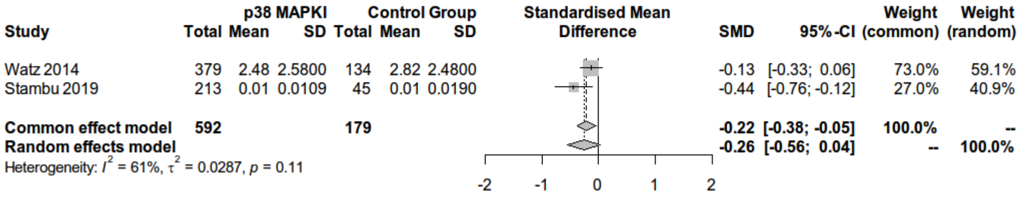

p = 0.09 (for random effect model)

2) Sensitivity analysis

There is only 2 studies included in this outcome, so there is no need for sensitivity analysis.

hsCRPratio

1) Forest plot

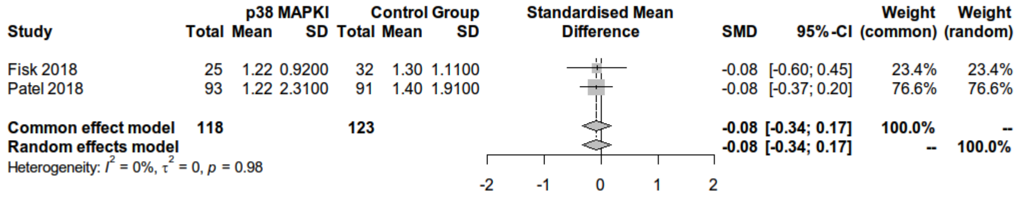

P = 0.52(for common effect model)

2) Sensitivity analysis

The I^2 is less than 50%, sensitivity analysis was not conducted for this outcome.

FIB

1) Forest plot

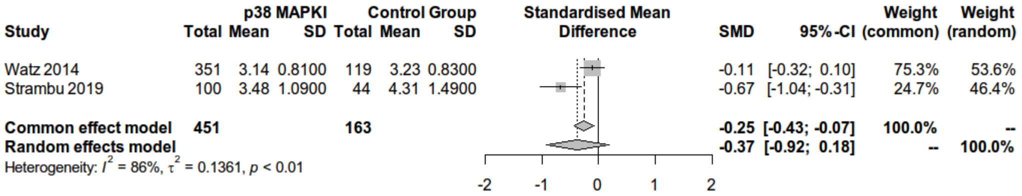

$p = 0.19$(for random effect model)

2) Sensitivity analysis

There is only 2 studies included in this outcome, so there is no need for sensitivity analysi

Section V. GRADE SOF table

Table 1: GRADE SOF of p38 MAPKIs' safety in COPD.

Summary of findings:

Safety of p38 MAPKI compared to placebo in COPD

Patient or population: COPD
Intervention: p38 MAPKI
Comparison: placebo

Outcomes	**Anticipated absolute effects*** (95% CI)		Relative effect (95% CI)	№ of participants (studies)	Certainty of the evidence (GRADE)	Comments
	**Risk with placebo**	**Risk with p38 MAPKI**				
Any adverse events (Overall AE)	679 per 1,000	**740 per 1,000** (652 to 842)	**RR 1.09** (0.96 to 1.24)	1134 (6 RCTs)	⊕⊕⊕⊕ High	P38 MAPKI does not increase any adverse events.
Severe adverse events (SAE)	83 per 1,000	**111 per 1,000** (55 to 226)	**RR 1.34** (0.66 to 2.72)	1287 (6 RCTs)	⊕⊕⊕◯ Moderate[a]	P38 MAPKI probably does not increase severe adverse events.
Exacerbation of COPD (ECOPD)	186 per 1,000	**197 per 1,000** (156 to 248)	**RR 1.06** (0.84 to 1.33)	1347 (7 RCTs)	⊕⊕⊕⊕ High	P38 MAPKI does not increase exacerbation of COPD.
Neurological adverse events	112 per 1,000	**85 per 1,000** (61 to 117)	**RR 0.76** (0.55 to 1.05)	1593 (9 RCTs)	⊕⊕⊕⊕ High	P38 MAPKI results in little to no difference in neurological adverse events.
Dental adverse events or adverse events occurred in ear, nose or throat. (DENT)	89 per 1,000	**100 per 1,000** (73 to 137)	**RR 1.13** (0.82 to 1.55)	1593 (9 RCTs)	⊕⊕⊕⊕ High	P38 MAPKI does not increase dental adverse events or adverse events occurred in ear, nose or throat. .

Summary of findings:

Safety of p38 MAPKI compared to placebo in COPD

Patient or population: COPD
Intervention: p38 MAPKI
Comparison: placebo

Outcomes	**Anticipated absolute effects*** (95% CI)		Relative effect (95% CI)	№ of participants (studies)	Certainty of the evidence (GRADE)	Comments
	**Risk with placebo**	**Risk with p38 MAPKI**				
Other respiratory infection	97 per 1,000	**119 per 1,000** (84 to 168)	**RR 1.22** (0.86 to 1.73)	1163 (7 RCTs)	⊕⊕⊕⊕ High	P38 MAPKI does not increase other respiratory infection.
Cardiovascular adverse events	16 per 1,000	**27 per 1,000** (11 to 66)	**RR 1.69** (0.69 to 4.11)	791 (7 RCTs)	⊕⊕⊕⊕ High	P38 MAPKI does not increase cardiovascular adverse events.
Digestive adverse events	79 per 1,000	**106 per 1,000** (64 to 177)	**RR 1.35** (0.81 to 2.25)	920 (4 RCTs)	⊕⊕⊕⊕ High	P38 MAPKI does not increase digestive adverse events.
Urinary infection	9 per 1,000	**28 per 1,000** (8 to 95)	**RR 3.29** (0.97 to 11.20)	905 (3 RCTs)	⊕⊕⊕⊕ High	P38 MAPKI does not increase urinary infection.
Musculoskeletal pain	124 per 1,000	**115 per 1,000** (87 to 152)	**RR 0.93** (0.70 to 1.23)	1332 (6 RCTs)	⊕⊕⊕⊕ High	P38 MAPKI does not reduce musculoskeletal pain.

*__The risk in the intervention group__ (and its 95% confidence interval) is based on the assumed risk in the comparison group and the **relative effect** of the intervention (and its 95% CI).

CI: confidence interval; **RR:** risk ratio

Summary of findings:

Safety of p38 MAPKI compared to placebo in COPD

Patient or population: COPD
Intervention: p38 MAPKI
Comparison: placebo

Outcomes	**Anticipated absolute effects*** (95% CI)		Relative effect (95% CI)	№ of participants (studies)	Certainty of the evidence (GRADE)	Comments
	**Risk with placebo**	**Risk with p38 MAPKI**				

GRADE Working Group grades of evidence
High certainty: we are very confident that the true effect lies close to that of the estimate of the effect.
Moderate certainty: we are moderately confident in the effect estimate: the true effect is likely to be close to the estimate of the effect, but there is a possibility that it is substantially different.
Low certainty: our confidence in the effect estimate is limited: the true effect may be substantially different from the estimate of the effect.
Very low certainty: we have very little confidence in the effect estimate: the true effect is likely to be substantially different from the estimate of effect.

Explanations
a. I^2 value > 50%.

Table 2: GRADE SOF of p38 MAPKIs' efficacy on COPD.

Summary of findings:

P38 MAPKI compared to placebo for improving the lung function, quality of life and inflammatory biomarker level in patients with COPD.

Patient or population: COPD
Intervention: p38 MAPKI
Comparison: placebo

Outcomes	Anticipated absolute effects* (95% CI)		Relative effect (95% CI)	№ of participants (studies)	Certainty of the evidence (GRADE)	Comments
	**Risk with placebo**	**Risk with p38 MAPKI**				
FEV1 pre-bronchodilator	-	SMD **0.13 SD more** (0.03 fewer to 0.3 more)	-	711 (4 RCTs)	⊕⊕⊕◯ Moderate[a]	P38 MAPKI probably does not increase FEV1 pre-bronchodilator.
FEV1 post-bronchodilator	-	SMD **0.11 SD more** (0.01 fewer to 0.24 more)	-	1190 (6 RCTs)	⊕⊕⊕⊕ High	P38 MAPKI does not increase FEV1 post-bronchodilator.
FVC pre-bronchodilator	-	SMD **0.07 SD more** (0.1 fewer to 0.23 more)	-	683 (3 RCTs)	⊕⊕⊕⊕ High	P38 MAPKI does not increase FVC pre-bronchodilator.

Summary of findings:

P38 MAPKI compared to placebo for improving the lung function, quality of life and inflammatory biomarker level in patients with COPD.

Patient or population: COPD
Intervention: p38 MAPKI
Comparison: placebo

Outcomes	**Anticipated absolute effects*** (95% CI)		Relative effect (95% CI)	№ of participants (studies)	Certainty of the evidence (GRADE)	Comments
	**Risk with placebo**	**Risk with p38 MAPKI**				
FVC post-bronchodilator	-	SMD **0.14 SD more** (0.01 more to 0.27 more)	-	1075 (4 RCTs)	⊕⊕⊕⊕ High	P38 MAPKI increases FVC post-bronchodilator slightly.
FEV1/FVC	-	SMD **0.42 SD more** (0.41 fewer to 1.25 more)	-	709 (2 RCTs)	⊕⊕⊕◯ Moderate[a]	P38 MAPKI probably does not increase FEV1/FVC.
TLC	-	SMD **0.08 SD fewer** (0.45 fewer to 0.3 more)	-	651 (2 RCTs)	⊕⊕⊕◯ Moderate[a]	P38 MAPKI probably results in little to no difference in TLC.
IC	-	SMD **0.07 SD more** (0.24 fewer to 0.39 more)	-	846 (3 RCTs)	⊕⊕⊕◯ Moderate[a]	P38 MAPKI probably does not increase IC.

Summary of findings:

P38 MAPKI compared to placebo for improving the lung function, quality of life and inflammatory biomarker level in patients with COPD.

Patient or population: COPD
Intervention: p38 MAPKI
Comparison: placebo

Outcomes	**Anticipated absolute effects*** (95% CI)		Relative effect (95% CI)	№ of participants (studies)	Certainty of the evidence (GRADE)	Comments
	**Risk with placebo**	**Risk with p38 MAPKI**				
SGRQ	-	SMD **0.04 SD fewer** (0.18 fewer to 0.1 more)	-	905 (4 RCTs)	⊕⊕⊕⊕ High	P38 MAPKI results in little to no difference in SGRQ.
hsCRP	-	SMD **0.26 SD lower** (0.56 lower to 0.04 higher)	-	771 (2 RCTs)	⊕⊕⊕◯ Moderate[a]	P38 MAPKI probably does not reduce hsCRP.
hsCRP ratio	-	SMD **0.08 SD lower** (0.34 lower to 0.17 higher)	-	241 (2 RCTs)	⊕⊕⊕⊕ High	P38 MAPKI results in little to no difference in hsCRP ratio.
FIB	-	SMD **0.37 SD lower** (0.92 lower to 0.18 higher)	-	614 (2 RCTs)	⊕⊕⊕◯ Moderate[a]	P38 MAPKI probably does not reduce FIB.

Summary of findings:

P38 MAPKI compared to placebo for improving the lung function, quality of life and inflammatory biomarker level in patients with COPD.

Patient or population: COPD
Intervention: p38 MAPKI
Comparison: placebo

Outcomes	Anticipated absolute effects* (95% CI)		Relative effect (95% CI)	№ of participants (studies)	Certainty of the evidence (GRADE)	Comments
	**Risk with placebo**	**Risk with p38 MAPKI**				

*__The risk in the intervention group__ (and its 95% confidence interval) is based on the assumed risk in the comparison group and the **relative effect** of the intervention (and its 95% CI).

CI: confidence interval; **SMD:** standardised mean difference
FEV1: forced expiratory volume in 1 second; **FVC**: forced vital capacity; **TLC**: total lung capacity; **IC**: inspiratory capacity; **SGRQ**: St Georges' respiratory questionnaire; **hsCRP**: high sensitivity C-reactive protein; FIB: fibrinogen

GRADE Working Group grades of evidence
High certainty: we are very confident that the true effect lies close to that of the estimate of the effect.
Moderate certainty: we are moderately confident in the effect estimate: the true effect is likely to be close to the estimate of the effect, but there is a possibility that it is substantially different.
Low certainty: our confidence in the effect estimate is limited: the true effect may be substantially different from the estimate of the effect.
Very low certainty: we have very little confidence in the effect estimate: the true effect is likely to be substantially different from the estimate of effect.

Explanations
a. I^2 value > 50%

Section VI. PRISMA 2020 Checklist

Section and Topic	Item #	Checklist item	Location where item is reported
**TITLE**			
Title	1	Identify the report as a systematic review.	Page 1
**ABSTRACT**			
Abstract	2	See the PRISMA 2020 for Abstracts checklist.	Page 1
**INTRODUCTION**			
Rationale	3	Describe the rationale for the review in the context of existing knowledge.	Page 2
Objectives	4	Provide an explicit statement of the objective(s) or question(s) the review addresses.	Page 2
**METHODS**			
Eligibility criteria	5	Specify the inclusion and exclusion criteria for the review and how studies were grouped for the syntheses.	Page 3
Information sources	6	Specify all databases, registers, websites, organisations, reference lists and other sources searched or consulted to identify studies. Specify the date when each source was last searched or consulted.	Page 3
Search strategy	7	Present the full search strategies for all databases, registers and websites, including any filters and limits used.	Page 2
Selection process	8	Specify the methods used to decide whether a study met the inclusion criteria of the review, including how many reviewers screened each record and each report retrieved, whether they worked independently, and if applicable, details of automation tools used in the process.	Page 3
Data collection process	9	Specify the methods used to collect data from reports, including how many reviewers collected data from each report, whether they worked independently, any processes for obtaining or confirming data from study investigators, and if applicable, details of automation tools used in the process.	Page 3
Data items	10a	List and define all outcomes for which data were sought. Specify whether all results that were compatible with each outcome domain in each study were sought (e.g. for all measures, time points, analyses), and if not, the methods used to decide which results to collect.	Page 3

Section and Topic	Item #	Checklist item	Location where item is reported
	10b	List and define all other variables for which data were sought (e.g. participant and intervention characteristics, funding sources). Describe any assumptions made about any missing or unclear information.	Page 3
Study risk of bias assessment	11	Specify the methods used to assess risk of bias in the included studies, including details of the tool(s) used, how many reviewers assessed each study and whether they worked independently, and if applicable, details of automation tools used in the process.	Page 3
Effect measures	12	Specify for each outcome the effect measure(s) (e.g. risk ratio, mean difference) used in the synthesis or presentation of results.	Page 3
Synthesis methods	13a	Describe the processes used to decide which studies were eligible for each synthesis (e.g. tabulating the study intervention characteristics and comparing against the planned groups for each synthesis (item #5)).	Page 3
	13b	Describe any methods required to prepare the data for presentation or synthesis, such as handling of missing summary statistics, or data conversions.	Page 3
	13c	Describe any methods used to tabulate or visually display results of individual studies and syntheses.	Page 3
	13d	Describe any methods used to synthesize results and provide a rationale for the choice(s). If meta-analysis was performed, describe the model(s), method(s) to identify the presence and extent of statistical heterogeneity, and software package(s) used.	Page 3
	13e	Describe any methods used to explore possible causes of heterogeneity among study results (e.g. subgroup analysis, meta-regression).	Page 3
	13f	Describe any sensitivity analyses conducted to assess robustness of the synthesized results.	Page 3
Reporting bias assessment	14	Describe any methods used to assess risk of bias due to missing results in a synthesis (arising from reporting biases).	Page 3
Certainty assessment	15	Describe any methods used to assess certainty (or confidence) in the body of evidence for an outcome.	Page 3

Section and Topic	Item #	Checklist item	Location where item is reported
**RESULTS**			
Study selection	16a	Describe the results of the search and selection process, from the number of records identified in the search to the number of studies included in the review, ideally using a flow diagram.	Page 3
	16b	Cite studies that might appear to meet the inclusion criteria, but which were excluded, and explain why they were excluded.	Page 3
Study characteristics	17	Cite each included study and present its characteristics.	Page 3
Risk of bias in studies	18	Present assessments of risk of bias for each included study.	Page 3
Results of individual studies	19	For all outcomes, present, for each study: (a) summary statistics for each group (where appropriate) and (b) an effect estimate and its precision (e.g. confidence/credible interval), ideally using structured tables or plots.	Page 4-5
Results of syntheses	20a	For each synthesis, briefly summarise the characteristics and risk of bias among contributing studies.	Page 4-5
	20b	Present results of all statistical syntheses conducted. If meta-analysis was done, present for each the summary estimate and its precision (e.g. confidence/credible interval) and measures of statistical heterogeneity. If comparing groups, describe the direction of the effect.	Page 4-5
	20c	Present results of all investigations of possible causes of heterogeneity among study results.	Page 3
	20d	Present results of all sensitivity analyses conducted to assess the robustness of the synthesized results.	Page 3
Reporting biases	21	Present assessments of risk of bias due to missing results (arising from reporting biases) for each synthesis assessed.	Page 4-5
Certainty of evidence	22	Present assessments of certainty (or confidence) in the body of evidence for each outcome assessed.	Page 4-5

Section and Topic	Item #	Checklist item	Location where item is reported
**DISCUSSION**			
Discussion	23a	Provide a general interpretation of the results in the context of other evidence.	Page 5
	23b	Discuss any limitations of the evidence included in the review.	Page 6
	23c	Discuss any limitations of the review processes used.	Page 6
	23d	Discuss implications of the results for practice, policy, and future research.	Page 6
**OTHER INFORMATION**			
Registration and protocol	24a	Provide registration information for the review, including register name and registration number, or state that the review was not registered.	Page 2
	24b	Indicate where the review protocol can be accessed, or state that a protocol was not prepared.	Page 2
	24c	Describe and explain any amendments to information provided at registration or in the protocol.	Page 2
Support	25	Describe sources of financial or non-financial support for the review, and the role of the funders or sponsors in the review.	Page 7
Competing interests	26	Declare any competing interests of review authors.	Page 7
Availability of data, code and other materials	27	Report which of the following are publicly available and where they can be found: template data collection forms; data extracted from included studies; data used for all analyses; analytic code; any other materials used in the review.	Page 7

From: Page MJ, McKenzie JE, Bossuyt PM, Boutron I, Hoffmann TC, Mulrow CD, et al. The PRISMA 2020 statement: an updated guideline for reporting systematic reviews. BMJ 2021;372:n71. doi: 10.1136/bmj.n71

For more information, visit: http://www.prisma-statement.org/
